# Supplementary material for: Identification and characterization of a large family of superbinding bacterial SH2 domains
Source: Nat Commun. 2018 Oct 31;9:4549. doi: 10.1038/s41467-018-06943-2 (PMC6208348; doi:10.1038/s41467-018-06943-2)
Supplement: Supplementary file 1 — Supplementary Information [file 41467_2018_6943_MOESM1_ESM.pdf]

Supplementary Information to
Identification and characterization of a large family of superbinding bacterial SH2 domains

Tomonori Kaneko[1], Peter J. Stogios[2], Xiang Ruan[1], Courtney Voss[1], Elena Evdokimova[2], Tatiana Skarina[2], Amy Chung[3], Xiaoling Liu[1], Lei Li[1,4], Alexei Savchenko[2,5], Alexander W. Ensminger[3], and Shawn S-C. Li[1,6,*]

[1]Department of Biochemistry, Schulich School of Medicine and Dentistry, Western University, London, Ontario N6G 2V4, Canada; [2]Department of Chemical Engineering and Applied Chemistry, University of Toronto, Toronto, Ontario M5G 1L6; [3]Departments of Biochemistry and Molecular Genetics, MaRS Centre, West Tower, Floor 16, Toronto, Ontario M5G 1M1; [4]Cancer institute, the Affiliated Hospital of Qingdao University, Qingdao Cancer Institute and School of Basic Medicine, Qingdao University, 266021, Qingdao, China; [5]Department of Microbiology, Immunology and Infectious Disease, University of Calgary, 2500 University Dr. NW, Calgary, Alberta T2N 1N4; [6]Children's Health Research Institute, Lawson Health Research Institute, 800 Commissioner's Road East, London, Ontario N6C 2V5, Canada.

*Corresponding author: sli@uwo.ca

Supplementary Information Contains 13 Supplementary Figures and 8 Supplementary Tables

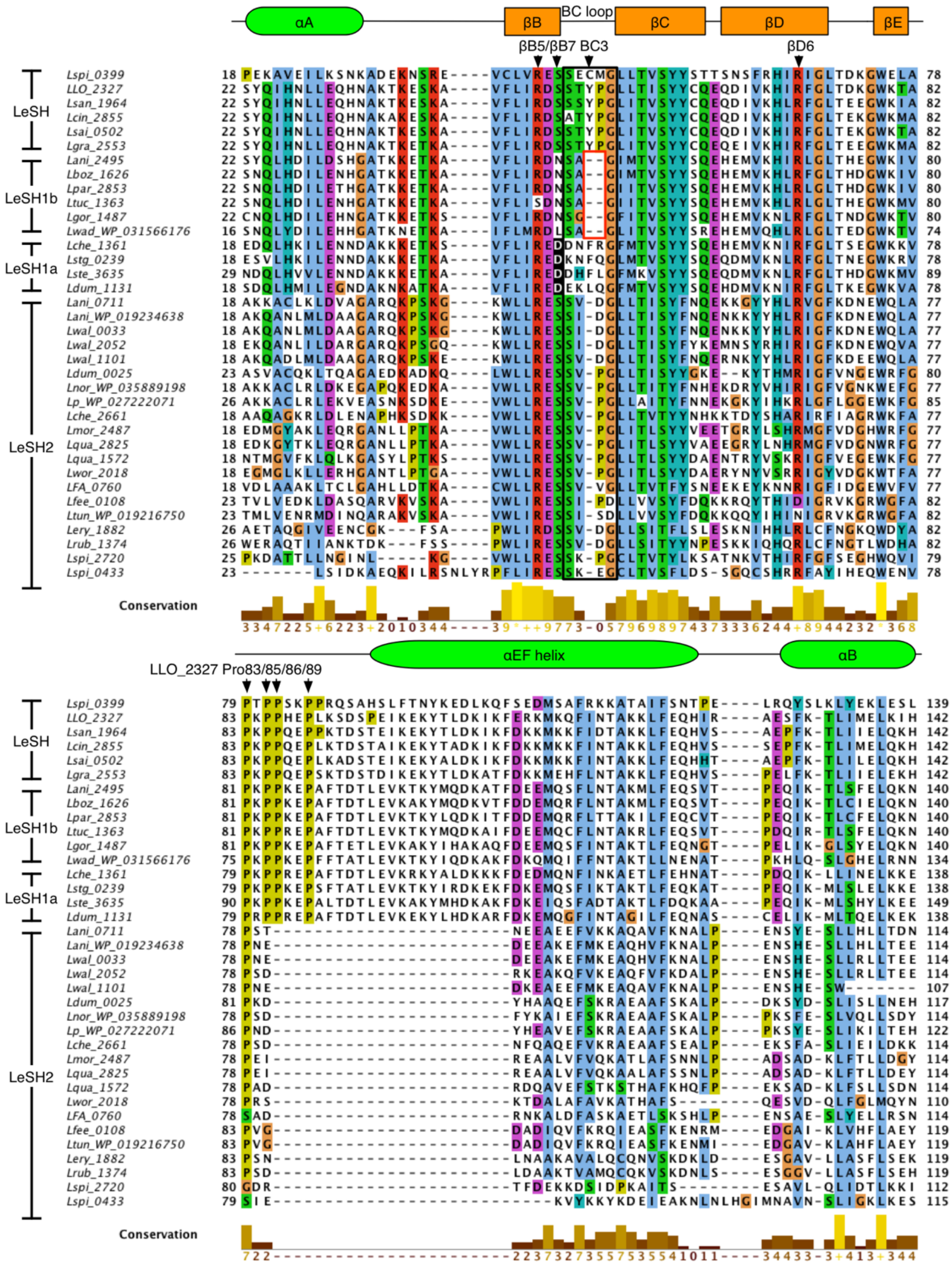

Supplementary Figure 1 | Sequence alignment of LeSH, LeSH1a, LeSH1b and LeSH2. The sequence alignment consists of six LeSH, four LeSH1a, six LeSH1b and 20 LeSH2 sequences (including paralogs LeSH2a, 2b, 2c). Arginine is strictly conserved at the βB5 position, except for the LeSH1b-like Ltuc_1363 that has serine at βB5. Arginine also occupies the βD6 position in all but two sequences. Four sequences have aspartic acid at the βB7 position (highlighted in black), which are classified as LeSH1a. The BC loop of LeSH1b is two residues shorter (the sequence gap in the red box) than that of LeSH and LeSH1a. The secondary structure elements are based on the LeSH structure. The figure was prepared using Jalview (http://www.jalview.org) with the "Clustalx" amino acid color assignment.

		LeSH						LeSH1b						LeSH1a				LeSH2																			
		Lspi_0399	LLO_2327	Lsan_1964	Lcin_2855	Lsai_0502	Lgra_2553	Lani_2495	Lboz_1626	Lpar_2853	Ltuc_1363	Lgor_1487	Lwad_WP_031566176	Lche_1361	Lstg_0239	Lste_3635	Ldum_1131	Lani_0711	Lani_WP_019234638	Lwal_0033	Lwal_2052	Lwal_1101	Ldum_0025	Lnor_WP_035889198	Lp_WP_027222071	Lche_2661	Lmor_2487	Lqua_2825	Lqua_1572	Lwor_2018	LFA_0760	Lfee_0108	Ltun_WP_019216750	Lery_1882	Lrub_1374	Lspi_2720	Lspi_0433
LeSH	Lspi_0399		33	31	31	32	31	34	30	31	31	30	29	34	32	27	31	37	34	34	36	37	33	33	31	33	31	30	29	32	29	30	30	23	22	30	29
	LLO_2327	33		83	83	87	79	55	55	55	52	51	46	51	53	50	54	32	34	34	34	33	35	35	32	33	32	32	28	34	28	29	28	24	24	31	26
	Lsan_1964	31	83		90	91	87	61	62	62	59	55	50	53	58	54	56	29	32	32	29	33	32	31	29	33	31	31	27	34	26	29	28	22	23	32	26
	Lcin_2855	31	83	90		91	86	59	59	59	57	54	49	52	55	52	56	27	30	30	29	30	31	31	28	32	29	29	26	32	25	29	29	21	22	32	24
	Lsai_0502	32	87	91	91		84	60	60	60	57	56	51	55	57	51	55	28	31	31	30	31	32	32	29	33	31	31	27	34	26	30	29	22	22	33	25
	Lgra_2553	31	79	87	86	84		65	64	62	62	57	52	55	56	53	58	28	29	29	28	32	32	32	30	33	31	31	28	34	27	29	28	20	22	31	25
LeSH1b	Lani_2495	34	55	61	59	60	65		90	88	89	76	67	67	69	66	65	29	30	30	28	30	28	29	26	28	27	27	26	29	27	28	26	18	17	23	24
	Lboz_1626	30	55	62	59	60	64	90		92	87	78	68	67	69	68	64	28	29	29	26	29	28	27	26	27	27	27	27	30	27	26	24	19	18	23	24
	Lpar_2853	31	55	62	59	60	62	88	92		87	76	70	64	67	64	63	28	29	29	26	29	28	27	27	27	28	28	28	32	27	26	24	20	18	22	23
	Ltuc_1363	31	52	59	57	57	62	89	87	87		76	69	67	66	64	62	28	29	29	25	29	27	26	26	26	29	29	25	29	26	26	24	19	17	21	23
	Lgor_1487	30	51	55	54	56	57	76	78	76	76		68	70	71	68	66	28	29	29	25	28	28	25	25	26	29	30	27	29	28	25	24	18	17	22	24
	Lwad_WP_031566176	29	46	50	49	51	52	67	68	70	69	68		56	61	55	53	27	28	27	26	28	29	27	26	28	29	29	28	28	30	25	23	20	19	21	21
LeSH1a	Lche_1361	34	51	53	52	55	55	67	67	64	67	70	56		75	69	70	25	27	27	26	26	27	26	26	29	31	32	27	28	29	28	28	17	20	25	27
	Lstg_0239	32	53	58	55	57	56	69	69	67	66	71	61	75		77	69	27	29	29	28	30	31	28	27	28	28	29	25	29	28	25	25	20	18	23	25
	Lste_3635	27	50	54	52	51	53	66	68	64	64	68	55	69	77		69	25	29	28	25	27	25	25	26	24	26	28	24	26	28	27	27	20	18	20	24
	Ldum_1131	31	54	56	56	55	58	65	64	63	62	66	53	70	69	69		25	28	28	29	29	26	26	26	27	28	30	24	28	26	29	29	20	20	25	26
LeSH2	Lani_0711	37	32	29	27	28	28	29	28	28	28	28	27	25	27	25	25		81	80	69	80	53	59	55	48	42	42	43	37	44	36	34	33	34	30	26
	Lani_WP_019234638	34	34	32	30	31	29	30	29	29	29	29	28	27	29	29	28	81		99	77	89	51	53	52	44	38	38	41	35	41	38	36	33	35	31	29
	Lwal_0033	34	34	32	30	31	29	30	29	29	29	29	27	27	29	28	28	80	99		78	89	51	53	52	44	38	38	41	35	41	37	35	33	35	31	29
	Lwal_2052	36	34	29	29	30	28	28	26	26	25	25	26	26	28	25	29	69	77	78		73	48	52	46	43	39	40	39	37	41	35	33	33	36	31	28
	Lwal_1101	37	33	33	30	31	32	30	29	29	29	28	28	26	30	27	29	80	89	89	73		52	54	54	48	38	37	40	37	41	37	34	31	33	31	28
	Ldum_0025	33	35	32	31	32	32	28	28	28	27	28	29	27	31	25	26	53	51	51	48	52		62	66	56	49	51	52	42	44	36	32	27	33	34	29
	Lnor_WP_035889198	33	35	31	31	32	32	29	27	27	26	25	27	26	28	25	26	59	53	53	52	54	62		69	62	44	45	48	41	42	37	35	29	31	33	26
	Lp_WP_027222071	31	32	29	28	29	30	26	26	27	26	25	26	26	27	26	26	55	52	52	46	54	66	69		59	40	42	45	34	39	32	32	29	33	34	26
	Lche_2661	33	33	33	32	33	33	28	27	27	26	26	28	29	28	24	27	48	44	44	43	48	56	62	59		39	39	44	37	41	38	37	27	30	36	27
	Lmor_2487	31	32	31	29	31	31	27	27	28	29	29	29	31	28	26	28	42	38	38	39	38	49	44	40	39		91	58	59	46	34	31	22	23	27	21
	Lqua_2825	30	32	31	29	31	31	27	27	28	29	30	29	32	29	28	30	42	38	38	40	37	51	45	42	39	91		56	57	48	35	32	23	26	26	22
	Lqua_1572	29	28	27	26	27	28	26	27	28	25	27	28	27	25	24	24	43	41	41	39	40	52	48	45	44	58	56		56	46	34	31	23	30	28	20
	Lwor_2018	32	34	34	32	34	34	29	30	32	29	29	28	28	29	26	28	37	35	35	37	37	42	41	34	37	59	57	56		40	30	28	22	21	26	22
	LFA_0760	29	28	26	25	26	27	27	27	27	26	28	30	29	28	28	26	44	41	41	41	41	44	42	39	41	46	48	46	40		29	27	31	29	26	26
	Lfee_0108	30	29	29	29	30	29	28	26	26	26	25	25	28	25	27	29	36	38	37	35	37	36	37	32	38	34	35	34	30	29		85	23	24	27	26
	Ltun_WP_019216750	30	28	28	29	29	28	26	24	24	24	24	23	28	25	27	29	34	36	35	33	34	32	35	32	37	31	32	31	28	27	85		27	27	24	26
	Lery_1882	23	24	22	21	22	20	18	19	20	19	18	20	17	20	20	20	33	33	33	33	31	27	29	29	27	22	23	23	22	31	23	27		68	22	24
	Lrub_1374	22	24	23	22	22	22	17	18	18	17	17	19	20	18	18	20	34	35	35	36	33	33	31	33	30	23	26	30	21	29	24	27	68		25	24
	Lspi_2720	30	31	32	32	33	31	23	23	22	21	22	21	25	23	20	25	30	31	31	31	31	34	33	34	36	27	26	28	26	26	27	24	22	25		34
	Lspi_0433	29	26	26	24	25	25	24	24	23	23	24	21	27	25	24	26	26	29	29	28	28	29	26	26	27	21	22	20	22	26	26	26	24	24	34	

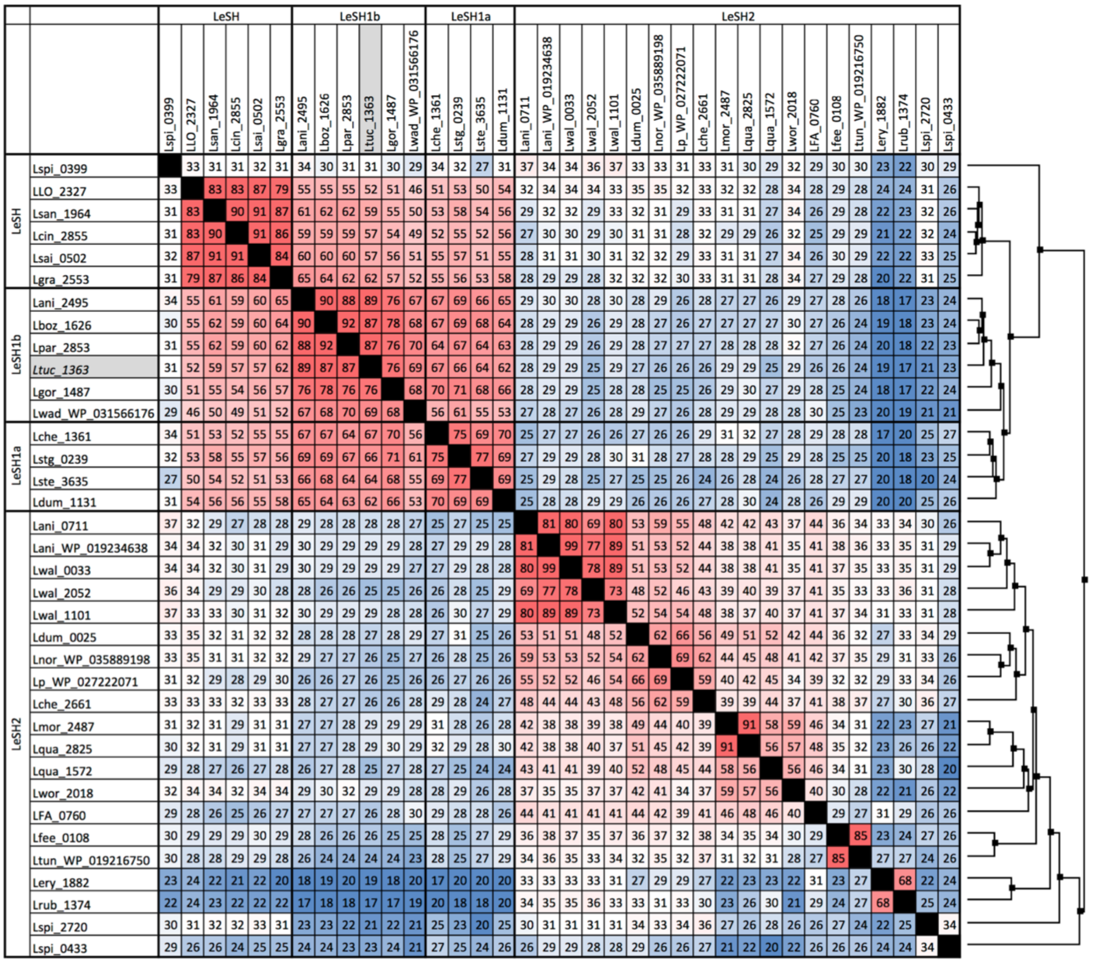

Supplementary Figure 2 | Sequence identities between LeSH, LeSH1a, LeSH1b and LeSH2 SH2 domains. Pairwise sequence identities between the domains are shown for all pairs, with a color gradient from low (blue) to high (red) identities. The phylogenetic tree on the right side shows relationship between the SH2 domains. The sequence identities and the average distance phylogenetic tree were derived from the sequence alignment in Supplementary Fig. 1. LeSH, LeSH1a, LeSH1b and LeSH2 belong to distinct phylogenetic branches.

a

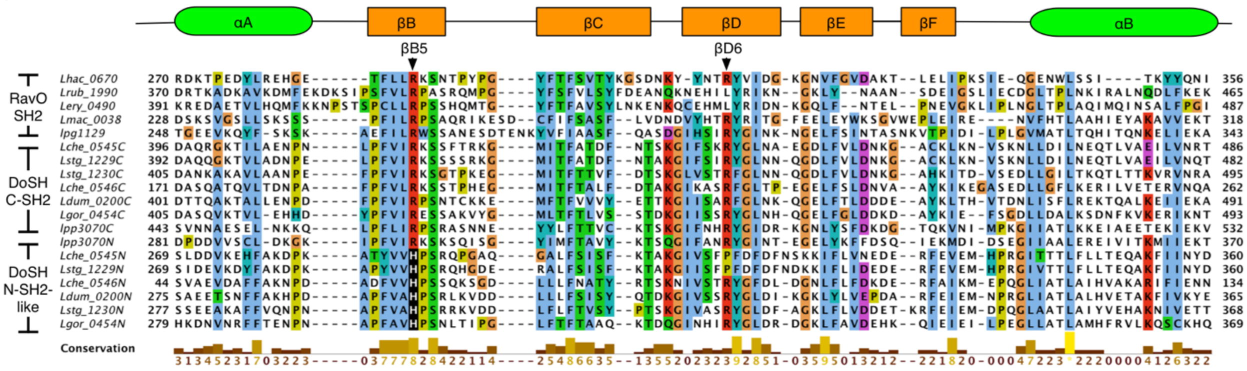

b

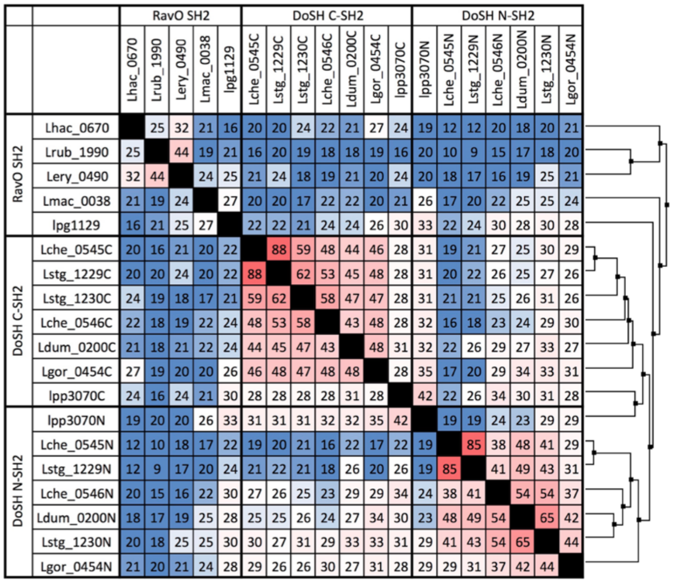

c

L. pneumophila strain	RavO	DoSH
Paris	LPP1130	LPP3070
Philadelphia-1	LPG1129	–
Thunder Bay	LP6_1111	–
Lorraine	LPO_1140	–
LPE509	LPE509_02042	–
HL06041035	LPV_1278	–
ATCC43290	LP12_1107	–
Alcoy	(fragments)	LPA_04394
Corby	(fragments)	LPC_3314
Lens	–	–

Supplementary Figure 3 | Sequence analysis of RavO and DoSH SH2 domains. a. Sequence alignment of five RavO SH2 domains and N- and C-terminal SH2 domains from seven DoSH (or DoSH1 and DoSH2) proteins. Note that a histidine, instead or an arginine, is located at βB5 (colored black in the alignment), in the N-terminal SH2 domain of the DoSH proteins except for *L. pneumophila* DoSH (lpp3070). The secondary structure elements are based on the structure of the RavO SH2 domain. **b.** Pairwise sequence identities between the SH2 domains and an average distance phylogenetic tree based on the alignment. **c.** Distribution of the genes encoding RavO and DoSH in *L. pneumophila* strains. The complete genome sequences of ten *L. pneumophila* strains were used for the analysis. The sequence identity between the equivalent proteins is at least 90%. The "–" symbol indicates that no homologous protein was found in the strain.

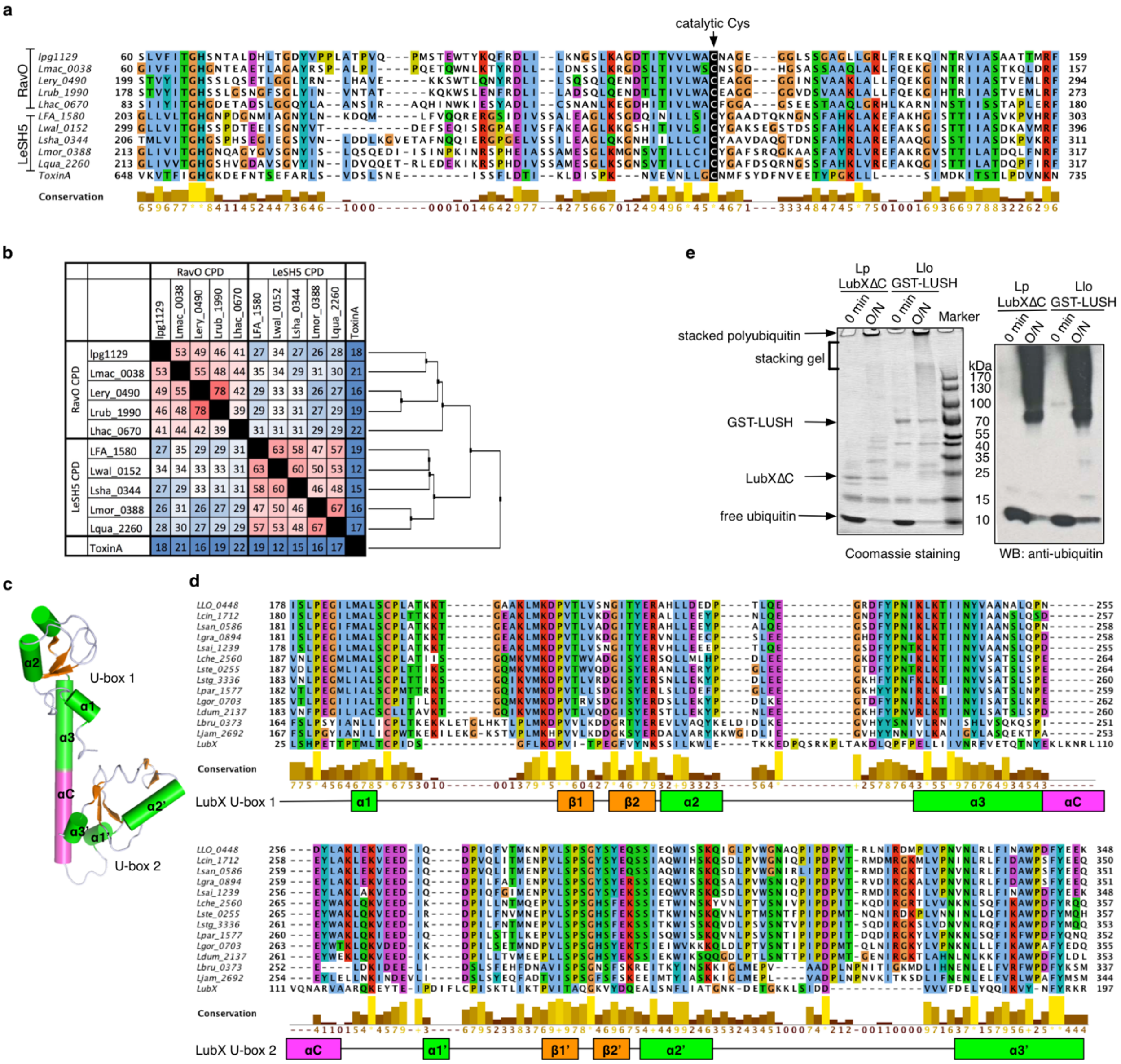

Supplementary Figure 4 | Conserved domains identified in *Legionella* effector proteins that contain an SH2 domain. a. Sequence alignment of six RavO cysteine protease domains (CPDs) and five LeSH5 CPDs. The cysteine protease Toxin A, a virulence factor secreted from *Clostridium difficile*, is included in the alignment as a reference. **b.** Pairwise sequence identities between the domains and an average distance phylogenetic tree based on the alignment in **a**. **c.** Structure of the *L. pneumophila* effector protein LubX, an E3 ubiquitin ligase with two U-box domains (PDB code 4WZ3). **d.** Sequence alignment of tandem U-box domains from 13 *Legionella* LUSH proteins. LubX is included as a reference for secondary structure elements. **e.** E3 ligase activity of *L. longbeachae* LUSH. Auto-ubiquitination assay was conducted using the full-length *L. longbeachae* LUSH fused with the GST tag (*Llo* GST-LUSH). Human UBE2D2 (also called UBCH5B) was used as the ubiquitin-conjugating enzyme E2. *L. pneumophila* LubX (with a C-terminal truncation, *Lp* LubXΔC, residue 1-186) was used as the positive control of the E3 ubiquitin ligase activity. O/N: overnight reaction.

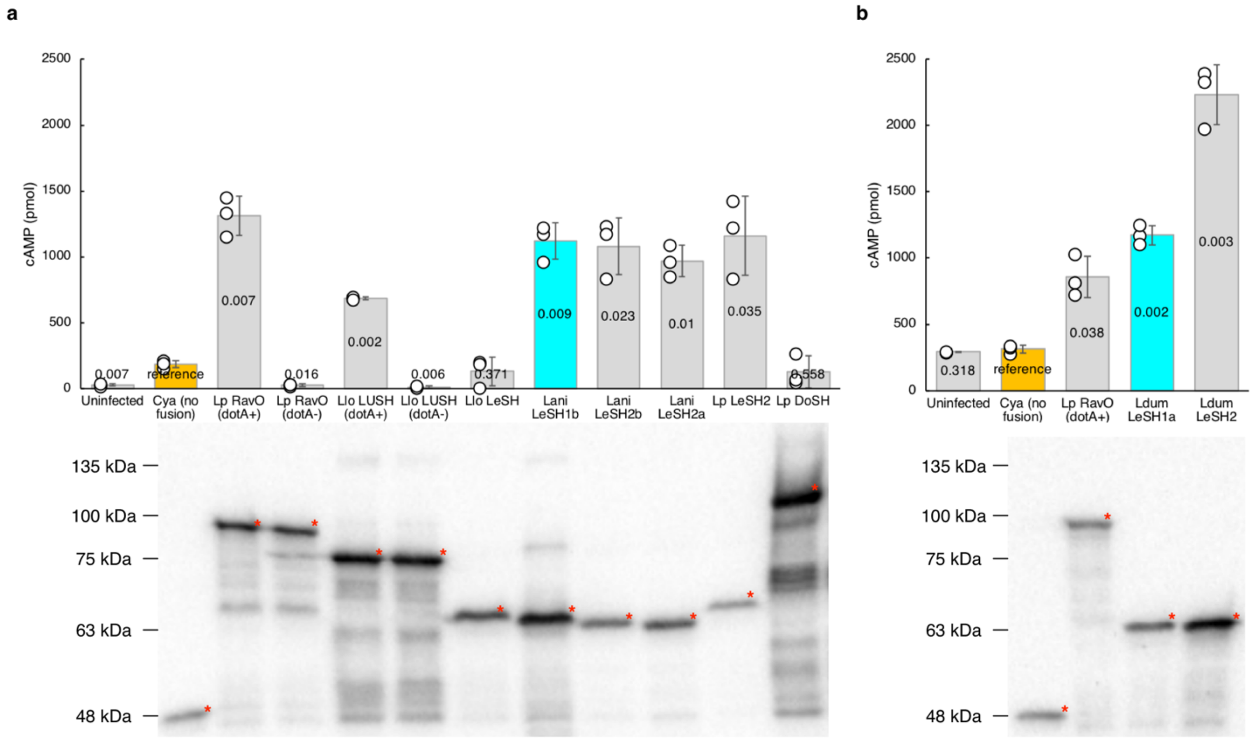

Supplementary Figure 5 | The effector translocation assay. a and **b**. *Legionella* SH2 proteins fused with the adenylate cyclase Cya were expressed in the *L. pneumophila* strain Lp02 (dotA+) or Lp03 (defect in the T4SS, or dotA-). Differentiated U937 cells were infected with bacteria expressing the different Cya-fusion protein. Translocation of the fusion proteins was monitored by the intracellular cyclic AMP (cAMP) level (upper panel), which is an indicator of the Cya activity in eukaryotic host cells. Protein expression level from each bacterial lysate was shown in the immunoblot (IB) using the antibody against Cya. The error bars indicate standard deviation from triplicate infection experiments. The two-tailed Student's paired t-test was employed to derive p-values that indicate statistically significant differences in translocation efficiency between each Cya-SH2 fusion protein and Cya alone ("no fusion", colored yellow), and the p-values were presented on each bar. Experiments in panels **a** and **b** were conducted on different days. The band of each Cya-fusion protein was indicated with a red asterisk (*) on the western blots. *L. anisa* LeSH1b and *L. dumoffii* LeSH1a, colored cyan, are inactive SH2 domain proteins with no binding to pTyr peptides (see Fig. 3c).

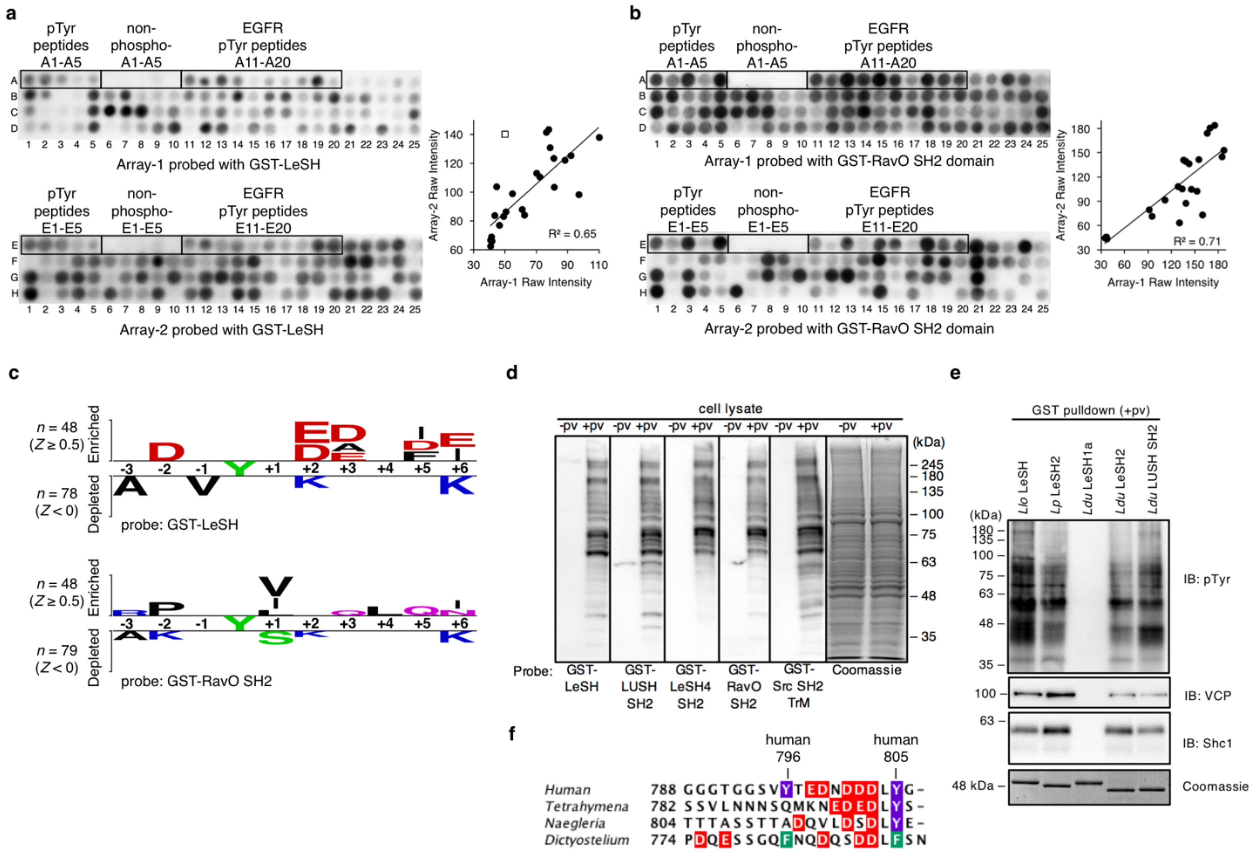

Supplementary Figure 6 | Binding of *Legionella* SH2 domains to tyrosine-phosphorylated peptides and proteins. a-b. Two copies of peptide array membranes (Array-1 and Array-2) incubated with either LeSH (panel a) or the RavO SH2 domain fused with GST (panel b). The first 20 peptides (A1 through A20) are identical in all four membranes. The bound protein was detected by the antibody against GST. See Supplementary Tables 3 and 4 for a list of peptide sequences and spot intensity readings. The correlation of spot intensities between Array-1 and Array-2 is also shown for LeSH (panel a), or for the RavO SH2 domain (panel b), derived from 24 pairs of identical peptides spotted in each membrane (spots A1 to A20 and four additional peptides). In panel a, one major outlier pair (open square), out of the 24 pairs, was excluded from the correlation calculation. The correlation was used for normalizing spot intensities between the two membranes to derive z-scores of peptide intensities (Supplementary Tables 5 and 6 for z-scores). **c.** Sequence preference of the *Legionella* SH2 domains. For each pair of membranes, either incubated with GST-LeSH (top panel) or the GST-RavO SH2 domain (bottom panel), peptides with high spot intensities (after normalization, $z \geq 0.5$) were used as the positive set, whereas those with $z < 0$ were used as the negative set, to generate the Two Sample Logo, based on the z-scores in Supplementary Tables 5 and 6. **d.** Far-Western blotting using *Legionella* SH2 domains to detect phosphoproteins. The same amount (20 µg) of U937 cell lysate, either with (+pv) or without (-pv) pervanadate treatment, was transferred to the PVDF membrane, and SH2 domains fused with GST were incubated with the membrane strips. The SH2 domain bound to the membrane was detected by an antibody against GST. **e.** GST pulldown assay that contains four *Legionella* SH2 domains not included in Fig. 3e. **f.** The C-terminal tail sequences of VCP orthologs in protozoan hosts. The protein sequences are derived from *Naegleria gruberi*

(GenBank EFC40318), *Tetrahymena thermophila* (GenBank EAR87202) and *Dictyostelium discoideum* (GenBank EAL63377). Acidic residues (Asp and Glu) are colored red, Tyr in purple, and Phe in green. Tyr796 and Tyr805 are the known phosphorylation sites of human VCP.

a

Fluorescein peptide	Sequence	*L. longbeachae* LeSH				*L. dumoffii* LeSH1a		*L. pneumophila* RavO SH2	
		wt	P85A	R46K	R71L	wt	D44S	wt	T310A
Shc1-pTyr239	G-G- D-H-Q-pY-Y-N-D-F-P-G -amide	7.1	15.0	>50	>50	>50	>50	0.2	1.5
Shc1-pTyr317	G-G- D-P-S-pY-V-N-V-Q-N-L -amide	6.7	11.9	>50	>50	>50	20.7	1.1	2.7
MidT-pTyr324	G-G- E-P-Q-pY-E-E-I-P-I-Y-L-amide	3.7	7.3	>50	>50	>50	45.4	6.6	11.2
GGpYGG	G-G-pY-G-G -amide	3.6	11.5	>50	>50	>50	>50	27.4	>50
IL2Rβ-pTyr387	G-G-Y-F-T-Y-D-P-pY-S-E-E-D-P-D -amide	0.9	3.7	4.0	>50	>50	21.1	7.6	24.2
DnaJ-A1-pTyr381	G-G-H-Y-N-G-E-A-pY-E-D-D-E-H-H -amide	0.5	1.6	3.0	>50	>50	>50	19.2	46.1
VCP-pTyr805-tail7	ahx- N-D-D-D-L-pY-G -COOH	2.2	5.9	43.2	>50	>50	>50	10.1	17.4
VCP-pTyr805-tail2	ahx- pY-G -COOH	1.0	4.3	>50	>50	>50	>50	9.4	21.0

ahx: 6-aminohexanoic acid linker

2.9 ± 0.9 fold reduction

K_D: 0.4 μM, 4 μM, 40 μM

2.9 ± 1.8 fold reduction (excluding GGpYGG peptide)

b

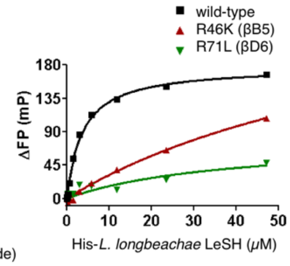

c

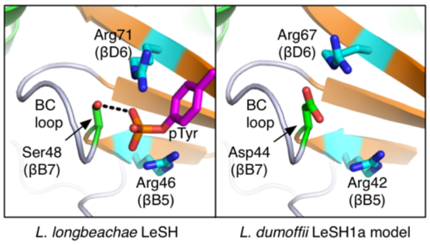

d

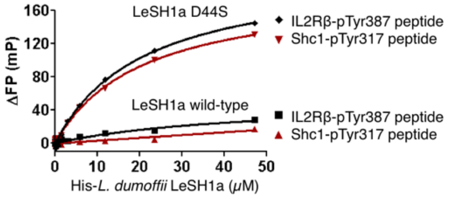

e

BC Length	human SH2
≤3	0
4	17
5	83
6	14
7	6
Total	120

f

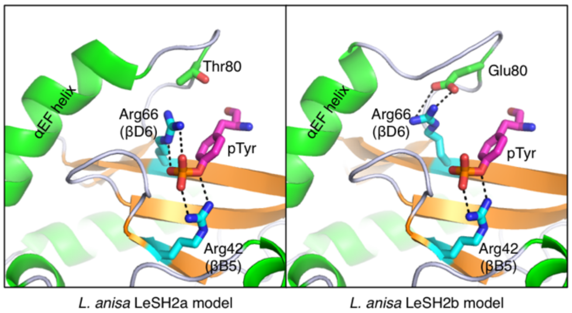

Supplementary Figure 7 | The phosphotyrosine binding pocket of *Legionella* SH2 domains. **a.** In-solution binding of phosphotyrosine-binding pocket mutants to tyrosine-phosphorylated peptides. Shown here are K_d values in μM, colored with a gradient from orange (high affinity) to blue (low affinity). The *Legionella* SH2 domains are N-terminally fused with a His_6 tag. **b.** In-solution binding of arginine mutants of His_6-tagged LeSH to the GGpYGG peptide. **c.** A model of *L. dumoffii* LeSH1a based on the *L. longbeachae* LeSH structure, highlighting the pTyr-binding pocket. Note that the βB7-Ser48 (shown as green sticks) in *L. longbeachae* LeSH is replaced by Asp44 in *L. dumoffi* LeSH1a. A potential salt bridge formed between Asp44 and two arginines Arg42 and Arg67 in the latter may compete off pTyr binding. **d.** The gain-of-function mutant LeSH1a D44S. Binding curves of the wild-type and D44S mutant of LeSH1a to two tyrosine-phosphorylated peptides are shown. **e.** BC loop length distribution of 120 human SH2 domains. The majority of the human SH2 domains have a five-residue BC loop. No human SH2 domain has a three-residue BC loop. **f.** Model structures of *L. anisa* LeSH2a (Lani_0711) and LeSH2b (Lani_WP_019234638) based on the LeSH structure. Both paralogs are encoded in the *L. anisa* Linanisette strain and they are 81% identical (Supplementary Fig. 2). Nevertheless, ligand binding activity of LeSH2b was substantially lower than that of LeSH2a (Fig. 3c). In the LeSH2b model, Glu80 is within the distance for an electric interaction with βD6 arginine (Arg66), which might reduce pTyr-binding activity of LeSH2b, compared to LeSH2a that has Thr80 at the equivalent position.

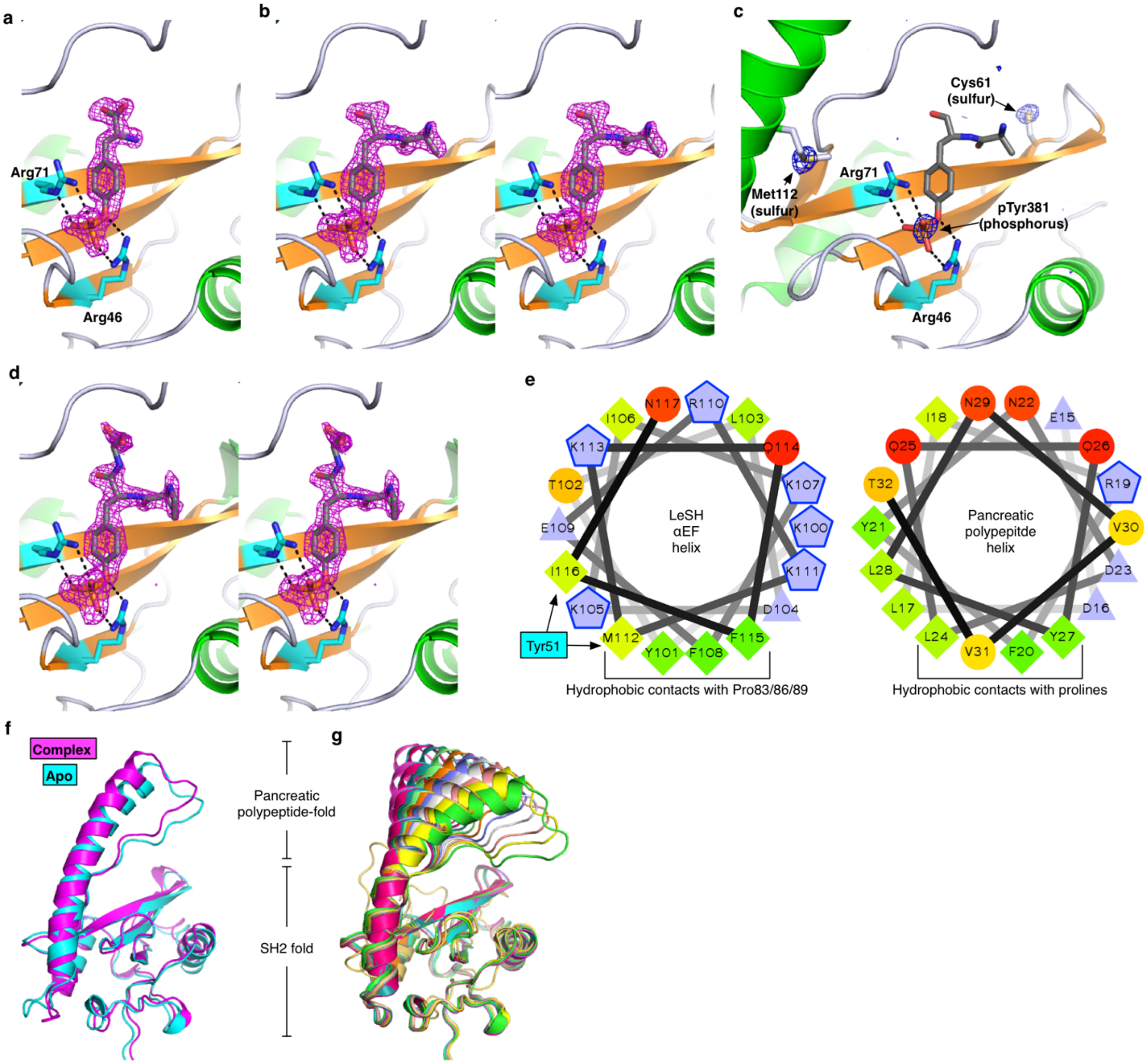

Supplementary Figure 8 | Crystallography of *L. longbeachae* LeSH. a. The *F*o-*F*c OMIT map of phosphotyrosine, contoured at 3 σ, in the complex structure of LeSH and the phosphotyrosine molecule. **b.** The electron density map of the DnaJ-A1 pTyr381 peptide HYNGEApY^{381}EDDEHH-amide bound to LeSH. Only the underlined two residues (Ala-pTyr) of the peptide were fit in the electron density. Wall-eyed stereo image of the *F*o-*F*c OMIT map calculated for the ligand peptide is contoured at 3 σ and shown as a magenta mesh. The two arginine residues that provide salt bridges to the phosphate group of phosphotyrosine are shown as cyan sticks, with H-bonds as dotted lines. **c.** Anomalous signals at the phosphotyrosine-binding site. Anomalous density peaks contoured at 4 σ shown as a blue mesh correspond to sulfur atoms of LeSH (in Cys61 and Met112 side chains) and the phosphorus atom of phosphotyrosine. **d.** The *F*o-*F*c OMIT map of the IL2Rβ pTyr387 peptide YFTYDPpY^{387}SEEDPD-amide bound to LeSH, contoured at 3 σ. Only the underlined three residues (Pro-pTyr-Ser) of the peptide were fit in the electron density. **e.** (*left*) Helical wheel projection for the αEF helix of LeSH. Positively charged residues (Arg and Lys) are shown as

blue pentagons. Pro83, Pro86 and Pro89 contact with the hydrophobic side of the helix. (*right*) Helical wheel projection of the avian pancreatic polypeptide (PDB code 2BF9). **f.** Comparison of the apo (cyan) and the ligand-bound (magenta) structures of LeSH. The IL2Rβ pTyr387 peptide complex structure was used here. The root-mean-square deviation value for the backbone is 1.0 Å. **g.** Normal mode analysis of the LeSH structure. Coordinates derived from the lowest frequency mode are superimposed for the SH2 domain fold. The elNémo server was used for calculation.

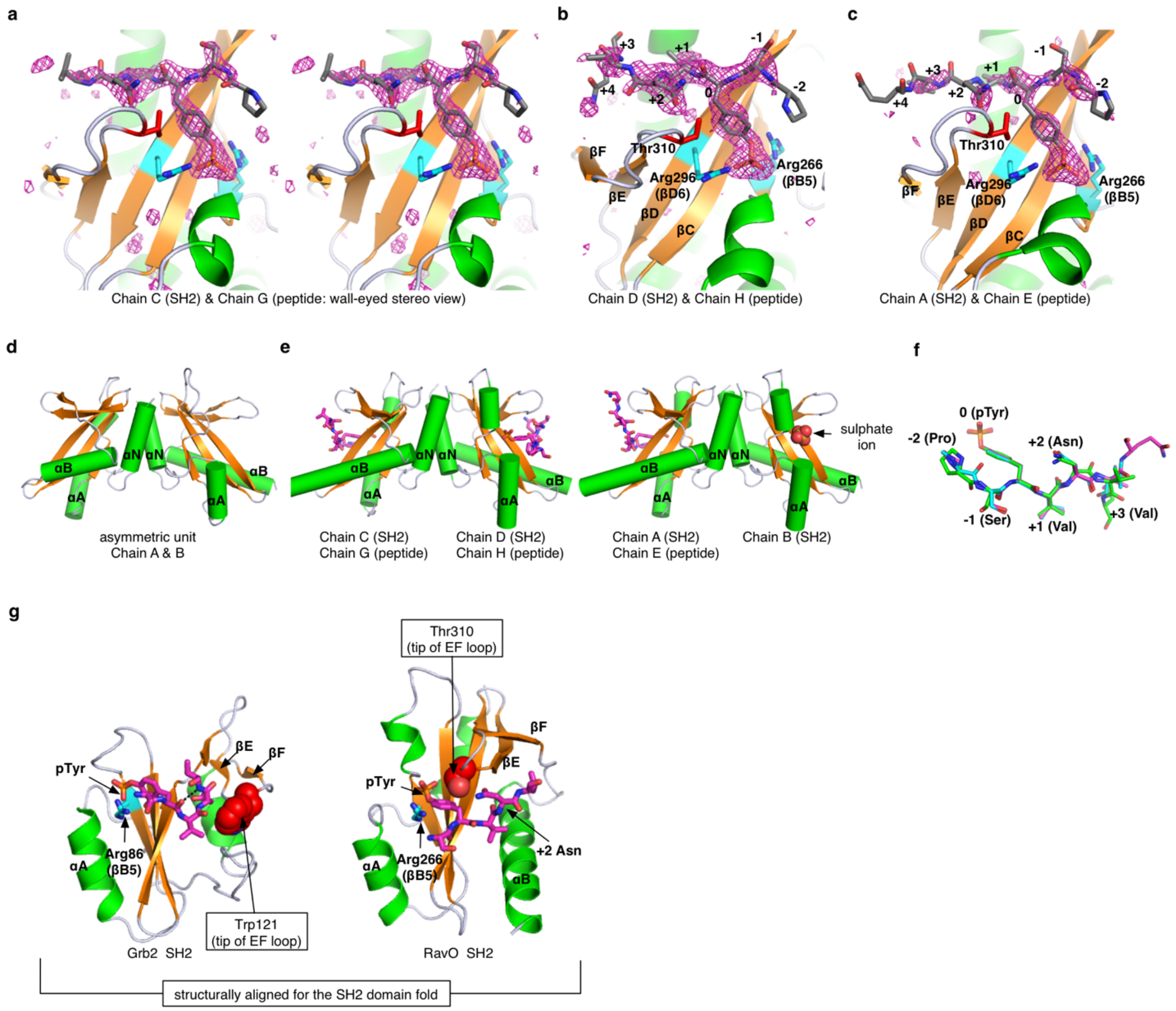

Supplementary Figure 9 | Crystallography of the RavO SH2 domain. a-c. The electron density maps of three Shc1 pTyr317 peptides identified in the asymmetric unit of the crystal of the SH2-peptide complex prepared by the peptide soaking method. The asymmetric unit consists of four SH2 molecules (chain A to D) and three peptide molecules (chain E, G and H). No bound peptide was observed for the SH2 molecule B, presumably due to crystal packing. The *F*o-*F*c OMIT map for the peptide contoured at 3 σ is shown as a magenta mesh. The two arginine residues (Arg266 and Arg296) are shown as cyan sticks. Thr310 is shown as red sticks. **a.** A wall-eyed stereo view of the complex between Chain C (RavO SH2) and Chain G (peptide). **b.** The complex between Chain D (RavO SH2) and Chain H (peptide). **c.** The complex between Chain A (RavO SH2) and Chain E (peptide). **d.** Two SH2 domain molecules in the asymmetric unit of the apo RavO SH2 domain crystal. **e.** Four RavO SH2 domains in the asymmetric unit of the crystal soaked with the peptide. The SH2 domains are arranged as two pairs of dimers due to the crystal contact, which was also observed in the apo crystal in panel d. No peptide was bound to Chain B of the SH2 domain but electron density assigned as a sulfate ion was observed in the pTyr-binding pocket. **f.** Overlay of the three Shc1 pTyr317 peptides bound to the RavO SH2 domain in the asymmetric unit. Chain E is in magenta, Chain G in cyan and Chain H in green. **g.**

Ribbon representation of the human Grb2 SH2 domain (PDB code 1JYR, left) in comparison with the *L. pneumophila* RavO SH2 domain (right) bound to the same Shc1 pTyr317 peptide. This panel corresponds to the surface representation in Fig. 6, e and f. Thr310 of the RavO SH2 domain and Trp121 of the Grb2 SH2 domain, each of them located at the tip of the EF loop, are highlighted as red spheres.

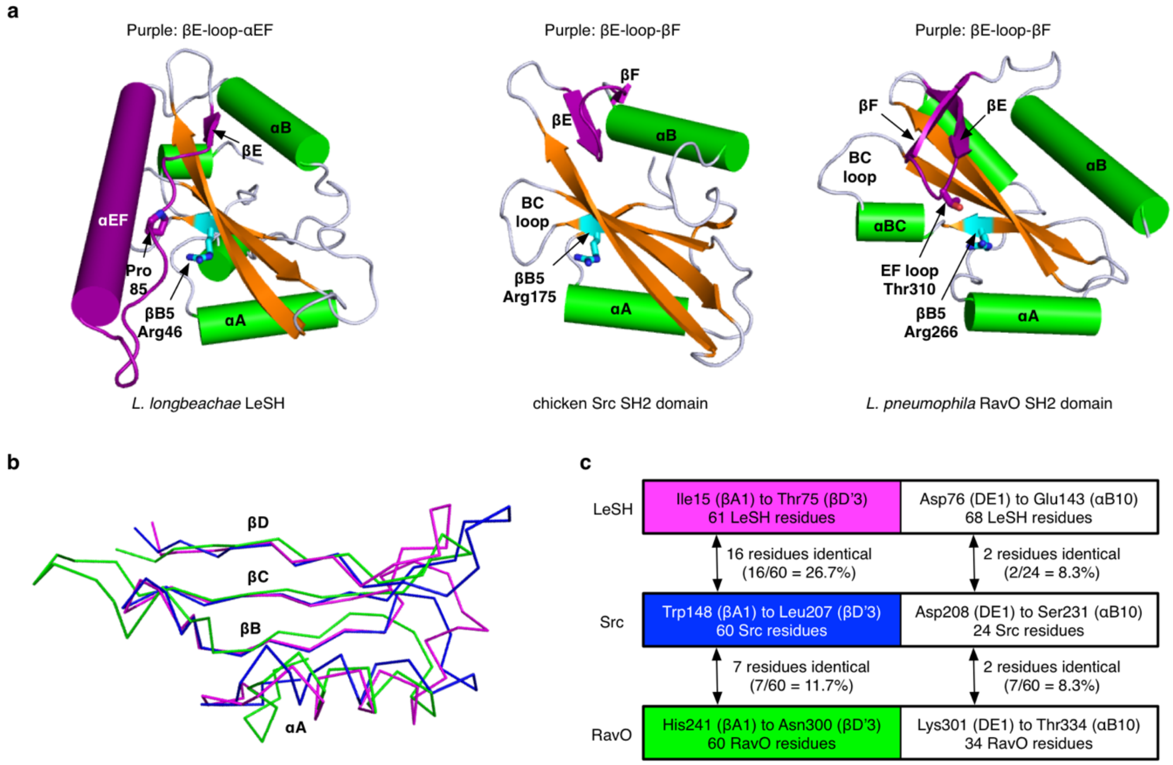

Supplementary Figure 10 | Comparison of the SH2 domain fold. a. Structures of *L. longbeachae* LeSH, the chicken Src SH2 domain and the *L. pneumophila* RavO SH2 domain are shown as ribbons. Helices and β-strands are colored green and orange, respectively. The region between βE and βF (or αEF for LeSH) is colored purple. The βB5 arginine is shown as cyan sticks. **b.** Backbone trace for the N-terminal half of the three SH2 domains. The region from αA to βD from each structure, involved in pTyr-binding pocket formation, is used for comparison. LeSH is in magenta, Src in blue and RavO in green. **c.** Sequence identities between the three structures, based on pairwise structure-based sequence alignment. Identical residues are counted for N-terminal (αA to βD) and C-terminal portions of the structures.

a

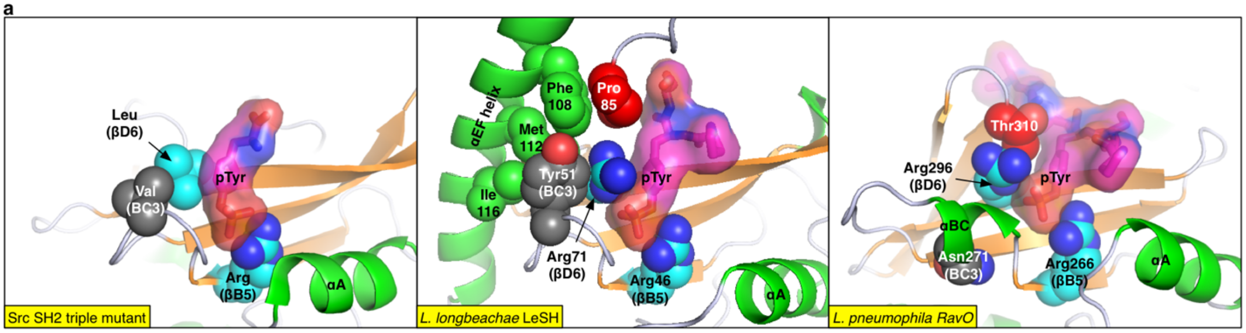

b

SH2 domain	Buried surface area for pTyr (Å^2)	PDB ID
**LeSH**	249	This work
PI3K p85α-C	229	5AUL
SOCS6	223	2VIF
SAP	214	1D4W
Cbl	212	3BUX
SHP2-C	208	5X7B
Src	205	1SPS
NCK2	205	2CIA
Fyn	202	4U1P
**RavO**	201	This work
Syk-N	196	1A81
PI3K p85α-N	191	2IUH
VAV2	189	4ROJ
BRDG1	185	3MAZ
Grb2	181	1JYR
SHP2-N	178	3TKZ
Syk-C	177	1A81

c

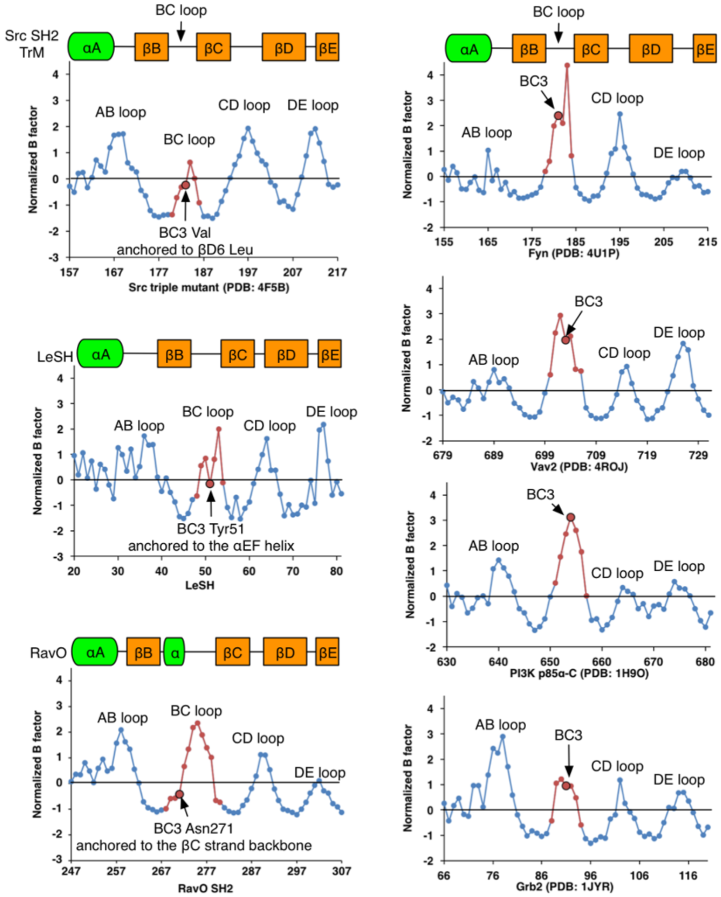

Supplementary Figure 11 | Structural comparison between *Legionella* and mammalian SH2 domains. a. A hydrophobic residue at the BC loop that stabilizes the loop in distinct ways. (*left* panel) The triple mutant of the Src SH2 domain (PDB code 4F5B). Valine at BC3 is paired with leucine at βD6 to form a hydrophobic contact and stabilizes the BC loop. The pair of hydrophobic residues synergistically increased binding affinity to phosphotyrosine. (*middle* panel) The phosphotyrosine binding pocket of LeSH. The BC3 residue Tyr51 is anchored to the hydrophobic patch provided by the amphiphatic helix. Tyr51 also stabilizes the βD6 arginine residue Arg71 that contributes to forming the salt bridge with pTyr. Note that the conformation of pTyr is different from Src SH2, as shown in Fig. 5b, thus the role of the βD6 is distinct between the two structures. (*right* panel) The phosphotyrosine binding pocket of the RavO SH2 domain. The region equivalent to the BC loop in the Src or LeSH SH2 domain (from BC1 to

BC4) is replaced by an α helix (which we named αBC), so that the local structure is stabilized by the defined secondary structure element. **b.** Buried surface area calculated for the pTyr residue in the ligand peptides bound to SH2 domains. LeSH (the IL2Rβ phosphopeptide complex), the RavO SH2 domain, and 15 human SH2 domain ligand complex structures were used here. **c.** Distribution of normalized B-factors of Cα atoms in SH2 domain crystals in complex with ligand peptides. The B-factor of an atom reflects the degree of structural variation (displacement) of the atom in the crystal. Flexible regions, such as loops, tend to be associated with higher B-factors. The B factors are normalized for the region between αA1 and βE4 as the Z-score. The four plots on the right panels were derived from wild type human SH2 domain structures complexed with ligand phosphopeptides. The three on the left are derived from the Src SH2 triple mutant (phosphotyrosine complex), LeSH (DnaJ-A1 peptide complex) and the RavO SH2 domain (Shc1 peptide complex).

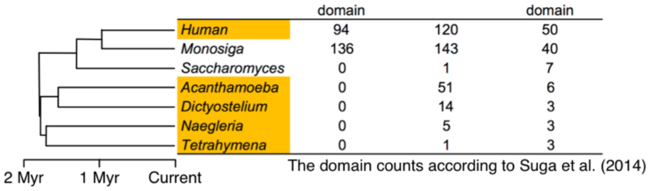

Species	Group A tyrosine kinase domain	SH2 domain	Tyrosine phosphatase domain
Human	94	120	50
Monosiga	136	143	40
Saccharomyces	0	1	7
Acanthamoeba	0	51	6
Dictyostelium	0	14	3
Naegleria	0	5	3
Tetrahymena	0	1	3

The domain counts according to Suga et al. (2014)

Supplementary Figure 12 | Distribution of phosphotyrosine signaling domains among eukaryotic host species of *Legionella*. The number of domains was taken from Suga et al. (*Mol Biol Evol* **31**, 517–528 (2014)). For example, human has 120 SH2 domains in 110 proteins. The time-calibrated phylogenetic tree was derived from Parfrey et al. (*Proc Natl Acad Sci USA* **108,** 13624–13629 (2011)). Myr: million year. Known host species of *Legionella* are highlighted in yellow. The species are *Monosiga brevicollis, Saccharomyces cerevisiae, Acanthamoeba castellanii, Dictyostelium discoideum, Naegleria gruberi, Tetrahymena thermophila*.

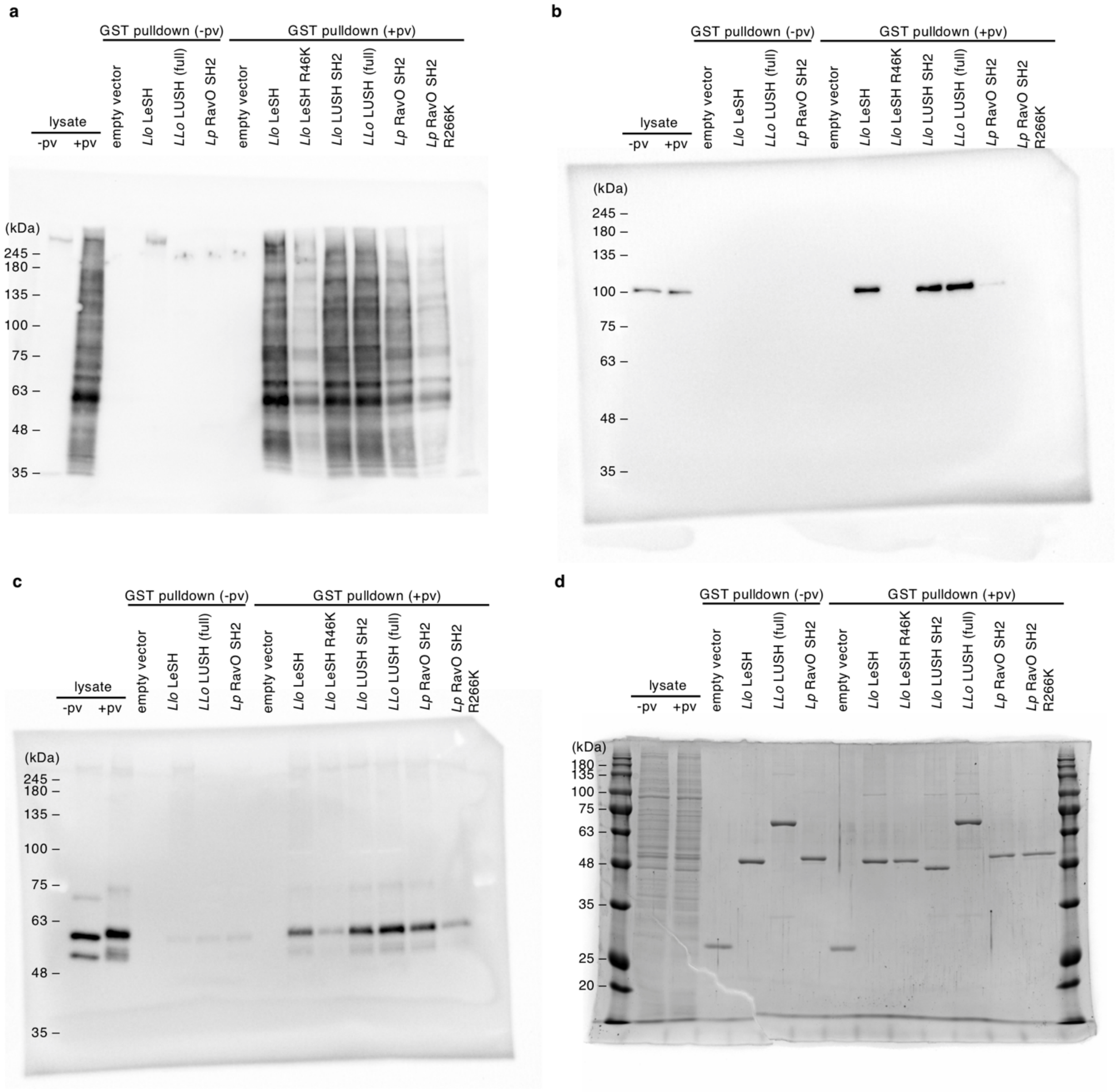

Supplementary Figure 13 | Uncropped scans of Western blot and Coomassie staining gels used in Fig. 3e. **a**. pTyr Western blot. **b**. VCP Western blot. **c**. Shc1 Western blot. **d**. Coomassie staining gel.

Supplementary Table 1. A list of bacterial proteins that contain a sequence profile for the SH2 domain.

NCBI #	Species	Strain	Identifier	Length	Name	βB5 residue number(s)	Other domains idefined by SMART and by Burstein et al.	LOG number (Burstein et al.)	Combined premissive learning score (taken from Burstein et al.)	Notes
KTC81785	*L. cincinnatiensis*	CDC#72-OH-14	Lcin_2855	168	LeSH	Arg46		LOG_02684	0.909	
KTD06610	*L. gratiana*	ATCC49413	Lgra_2553	167	LeSH	Arg46		LOG_02684	0.911	
CBJ12739	*L. longbeachae*	NSW150	LLO_2327	167	LeSH	Arg46		LOG_02684	0.966	
KTD59858	*L. sainthelensi*	Mt.St.Helens-4	Lsai_0502	167	LeSH	Arg46		LOG_02684	0.983	
KTD60673	*L. santicrucis*	SC-63-C7	Lsan_1964	167	LeSH	Arg46		LOG_02684	0.996	
KTD65687	*L. spiritensis*	Mt.St.Helens-9	Lspi_0399	181	LeSH	Arg42		LOG_02684	0.987	
KTC79341	*L. cherrii*	ORW	Lche_1361	189	LeSH1a	Arg42		LOG_02684	0.896	
KTC90063	*L. dumoffii*	NY-23	Ldum_1131	182	LeSH1a	Arg42		LOG_02684	0.996	
KTD67429	*L. steelei*	IMVS3376	Lste_3635	188	LeSH1a	Arg53		LOG_02684	0.900	
KTD81012	*L. steigerwaltii*	SC-18-C9	Lstg_0239	189	LeSH1a	Arg42		LOG_02684	0.993	
KTD41536	*L. parisiensis*	PF-209-C-C2	Lpar_2853	184	LeSH1b	Arg46		LOG_02684	0.936	
KTC69994	*L. anisa*	WA-316-C3	Lani_2495	184	LeSH1b	Arg46		LOG_02684	0.923	NCBI# WP_019233116 in the Linanisette strain
KTC74186	*L. bozemanii*	WIGA	Lboz_1626	184	LeSH1b	Arg46		LOG_02684	0.878	
KTD03502	*L. gormanii*	LS-13	Lgor_1487	180	LeSH1b	Arg46		LOG_02684	0.957	
WP_031566176	*L. wadsworthii*	ATCC 33877	Lwad_WP_031566176	177	LeSH1b	Arg40		N/A	N/A	
KTD73516	*L. tucsonensis*	ATCC49180	Ltuc_1363	184	LeSH1b-like	*Ser46*		LOG_02684	0.860	Ser46 at βB5
KTC74912	*L. anisa*	WA-316-C3	Lani_0711	155	LeSH2a	Arg42		LOG_02859	0.998	NCBI# WP_019234449 in the Linanisette strain
WP_019234638	*L. anisa*	Linanisette	Lani_WP_019234638	155	LeSH2b	Arg42		N/A	N/A	
KTC80641	*L. cherrii*	ORW	Lche_2661	157	LeSH2	Arg42		LOG_02859	0.997	
KTC93505	*L. dumoffii*	NY-23	Ldum_0025	159	LeSH2	Arg47		LOG_02859	0.991	
KTC96090	*L. erythra*	SE-32A-C8	Lery_1882	156	LeSH2	Arg47		LOG_02859	0.991	
WP_052673840	*L. fallonii*	LLAP-10	LFA_0760	153	LeSH2	Arg42		N/A	N/A	
KTD04650	*L. feeleii*	WO-44C	Lfee_0108	157	LeSH2	Arg47		LOG_02859	0.973	
KTD31611	*L. moravica*	ATCC43877	Lmor_2487	193	LeSH2	Arg42		LOG_02859	0.993	
WP_035889198	*L. norrlandica*	LEGN	Lnor_WP_035889198	156	LeSH2	Arg42		N/A	N/A	
WP_027222071	*L. pneumophila*	ATCC 33737	Lp_WP_027222071	164	LeSH2	Arg50		N/A	N/A	
KTD51345	*L. quateirensis*	ATCC49507	Lqua_1572	159	LeSH2a/2b	Arg42		LOG_02859	0.999	
KTD44658	*L. quateirensis*	ATCC49507	Lqua_2825	193	LeSH2a/2b	Arg42		LOG_02859	0.996	
KTD49023	*L. rubrilucens*	WA-270A-C2	Lrub_1374	152	LeSH2	Arg47		LOG_02859	0.990	
KTD65721	*L. spiritensis*	Mt.St.Helens-9	Lspi_0433	266	LeSH2a/2b	Arg44		LOG_02859	1.000	
KTD61100	*L. spiritensis*	Mt.St.Helens-9	Lspi_2720	195	LeSH2a/2b	Arg44		LOG_02859	1.000	
WP_019216750	*L. tunisiensis*	LegM	Ltun_WP_019216750	121	LeSH2	Arg47		N/A	N/A	
KTD83125	*L. waltersii*	ATCC51914	Lwal_0033	155	LeSH2a/2b/2c	Arg42		LOG_02859	1.000	
KTD80404	*L. waltersii*	ATCC51914	Lwal_1101	107	LeSH2a/2b/2c	Arg42		LOG_02684	0.986	
KTD76330	*L. waltersii*	ATCC51914	Lwal_2052	155	LeSH2a/2b/2c	Arg42		LOG_02859	1.000	
KTD76793	*L. worsleiensis*	ATCC49508	Lwor_2018	166	LeSH2	Arg42		LOG_02859	0.991	
KTC89484	*L. dumoffii*	NY-23	Ldum_0552	320	LeSH3	Arg55		LOG_07829	0.040	
KTD00235	*L. gormanii*	LS-13	Lgor_3130	332	LeSH3	Arg55		LOG_07829	0.056	
WP_031567682	*L. wadsworthii*	ATCC 33877	Lwad_WP_031567682	298	LeSH3	Arg36		N/A	N/A	
KTC73067	*L. birminghamensis*	CDC#1407-AL-14	Lbir_1119	352	LeSH4	Arg62	Ubox (254-300)	LOG_04518	1.000	
KTC93200	*L. cincinnatiensis*	CDC#72-OH-14	Lcin_0238	453	LeSH4	Arg109		LOG_04518	1.000	
EHL28955	*L. drancourtii*	LLAP12	LDG_8949	391	LeSH4	Arg66		LOG_04518	0.998	
KTD14725	*L. israelensis*	Bercovier 4	Lisr_2433	296	LeSH4	Arg50		LOG_04518	0.998	
KTD20948	*L. lansingensis*	ATCC49751	Llan_1678	423	LeSH4	Arg94		LOG_04518	1.000	
WP_043873924	*L. massiliensis*	LegA	Lmas_WP_043873924	351	LeSH4	Arg58		N/A	N/A	
WP_058535099	*L. saoudiensis*	LH-SWC	Lsao_WP_058535099	392	LeSH4	Arg53		N/A	N/A	
KTD50084	*L. quinlivanii*	CDC#1442-AUS-E	Lqui_1409	373	LeSH4	Arg60	Ubox (263-307)	LOG_04518	1.000	
WP_045095561	*L. fallonii*	LLAP-10	LFA_1580	601	LeSH5	Arg49		N/A	N/A	
KTD37525	*L. moravica*	ATCC43877	Lmor_0388	587	LeSH5	Arg50	CPD (209-317), L06 (510-582)	LOG_04812	1.000	
KTD47866	*L. quateirensis*	ATCC49507	Lqua_2260	436	LeSH5	Arg50	CPD (209-317)	LOG_04812	0.999	
KTD64975	*L. shakespearei*	ATCC49655	Lsha_0344	547	LeSH5	Arg49	CPD (202-311), L06 (459-538)	LOG_04812	1.000	
KTD83036	*L. waltersii*	ATCC51914	Lwal_0152	533	LeSH5	Arg51, Arg155	CPD (295-396)	LOG_04812	1.000	
KTC86432	*L. brunensis*	ATCC43878	Lbru_0373	344	LUSH	Arg55	Ubox (174-242), Ubox (259-323)	LOG_02977	0.294	
KTC80540	*L. cherrii*	ORW	Lche_2560	375	LUSH	Arg60	Ubox (276-350)	LOG_02977	0.304	
KTC86252	*L. cincinnatiensis*	CDC#72-OH-14	Lcin_1712	361	LUSH	Arg56	Ubox (192-246), Ubox (272-343)	LOG_02977	0.409	
KTC91069	*L. dumoffii*	NY-23	Ldum_2137	368	LUSH	Arg56	Ubox (272-346)	LOG_02977	0.608	
KTD05072	*L. gormanii*	LS-13	Lgor_0703	369	LUSH	Arg58	Ubox (274-348)	LOG_02977	0.465	
KTD13602	*L. gratiana*	ATCC49413	Lgra_0894	362	LUSH	Arg56	Ubox (193-247), Ubox (273-344)	LOG_02977	0.423	
KTD08497	*L. jamestowniensis*	JA-26-G1-E2	Ljam_2692	353	LUSH	Arg54	Ubox (267-330)	LOG_02977	0.351	
CBJ10779	*L. longbeachae*	NSW150	LLO_0448	359	LUSH	Arg56	Ubox (190-244), Ubox (270-341)	LOG_02977	0.705	
KTD40260	*L. parisiensis*	PF-209-C-C2	Lpar_1577	367	LUSH	Arg59	Ubox (274-345)	LOG_02977	0.455	
KTD58632	*L. sainthelensi*	Mt.St.Helens-4	Lsai_1239	359	LUSH	Arg56	Ubox (190-244), Ubox (270-341)	LOG_02977	0.796	
KTD66641	*L. santicrucis*	SC-63-C7	Lsan_0586	362	LUSH	Arg56	Ubox (193-247), Ubox (273-344)	LOG_02977	0.431	
KTD71489	*L. steelei*	IMVS3376	Lste_0255	375	LUSH	Arg60	Ubox (276-350)	LOG_02977	0.446	
KTD69895	*L. steigerwaltii*	SC-18-C9	Lstg_3336	371	LUSH	Arg56	Ubox (272-346)	LOG_02977	0.299	
KRG18663	*Coxiellaceae* bacterium	HT99	Coxi_KRG18663	343	LUSH	Arg49	Ubox (260-316)	N/A	N/A	
KTC99589	*L. erythra*	SE-32A-C8	Lery_0490	655	RavO	Arg415	CPD (195-294), L35 (568-625)	LOG_05208	0.999	
KTD13826	*L. hackeliae*	798-PA-H	Lhac_0670	371	RavO	Arg288	CPD (79-180)	LOG_05208	0.999	
KTD31994	*L. maceachernii*	PX-1-G2-E2	Lmac_0038	354	RavO	Arg246	CPD (56-157)	LOG_05208	1.000	
AAU27215	*L. pneumophila*	Philadelphia-1	lpg1129	519	RavO	Arg266	CPD (56-159)	LOG_05208	1.000	lpp1130 in the Paris strain
KTD47068	*L. rubrilucens*	WA-270A-C2	Lrub_1990	634	RavO	Arg394	CPD (174-273), L35 (542-598)	LOG_05208	0.999	
KTC82281	*L. cherrii*	ORW	Lche_0545	538	DoSH1	Arg415	L22 (58-200)	LOG_00141	0.997	
KTC82282	*L. cherrii*	ORW	Lche_0546	317	DoSH2	Arg190		LOG_08605	0.994	
KTC92394	*L. dumoffii*	NY-23	Ldum_0200	546	DoSH	Arg420	L22 (59-206)	LOG_00141	0.997	
KTD05240	*L. gormanii*	LS-13	Lgor_0454	548	DoSH	Arg423	L22 (58-208)	LOG_00141	0.996	
CAH14223	*L. pneumophila*	Paris	lpp3070	586	DoSH	Arg299, Arg461		N/A	N/A	
KTD78494	*L. steigerwaltii*	SC-18-C9	Lstg_1229	533	DoSH1	Arg411	L22 (58-200)	LOG_00141	0.999	
KTD78495	*L. steigerwaltii*	SC-18-C9	Lstg_1230	548	DoSH2	Arg424	L22 (59-206)	LOG_00141	0.997	
KTC78335	*L. brunensis*	ATCC43878	Lbru_2627	1013	out-group	Arg398, Arg608	L60 (5-108), L57 (189-312)	LOG_04006	1.000	
KTD28480	*L. micdadei*	TATLOCK	Lmic_1591	185	out-group	Arg61		LOG_11994	0.681	
KTD30434	*L. maceachernii*	PX-1-G2-E2	Lmac_0618	197	out-group	Arg78		LOG_02977	0.010	
KTD45217	*L. quinlivanii*	CDC#1442-AUS-E	Lqui_2688	154	out-group	Arg61		LOG_13428	0.648	
KTD82717	*L. waltersii*	ATCC51914	Lwal_0459	99	out-group	Arg65		LOG_14988	0.757	
WP_057624523	*Coxiellaceae* bacterium	CC99	Coxi_WP_057624523	283	out-group	Arg53		N/A	N/A	

Supplementary Table 2. Curve fitting stitistics of in-solution peptide binding experiments

	L. pneumophila His-RavO SH2	*L. longbeachae* His-LeSH	*L. longbeachae* His-LUSH SH2	*L. pneumophila* His-DoSH C-SH2	*L. dumoffii* LUSH (full length)	*L. drancourtii* His-LeSH4 SH2
Shc1-pTyr239	0.22 ± 0.02	7.15 ± 0.71	0.30 ± 0.03	0.40 ± 0.02	0.13 ± 0.01	0.55 ± 0.04
Shc1-pTyr317	1.14 ± 0.09	6.71 ± 0.38	0.68 ± 0.08	0.64 ± 0.04	0.40 ± 0.04	1.58 ± 0.11
EGFR-pTyr954	3.66 ± 0.54	>50	0.57 ± 0.03	0.15 ± 0.01	0.95 ± 0.04	1.35 ± 0.14
EGFR-pTyr1086	7.06 ± 0.65	20.37 ± 2.15	1.64 ± 0.11	12.49 ± 0.78	0.18 ± 0.04	1.04 ± 0.12
MidT-pTyr324	6.59 ± 0.54	3.70 ± 0.29	0.41 ± 0.04	32.01 ± 7.66	0.37 ± 0.02	1.59 ± 0.15
GGpYGG	27.39 ± 2.77	3.61 ± 0.31	0.36 ± 0.04	27.36 ± 3.97	0.38 ± 0.05	0.13 ± 0.01
IL2Rβ-pTyr387	7.57 ± 0.46	0.91 ± 0.06	0.36 ± 0.06	22.96 ± 1.53	0.28 ± 0.02	2.00 ± 0.22
DnaJ-A1-pTyr381	19.21 ± 2.73	0.53 ± 0.08	0.24 ± 0.03	>50	0.35 ± 0.02	0.81 ± 0.18
DnaJ-A1-pTyr381ΔN	17.64 ± 1.12	1.61 ± 0.10	0.25 ± 0.02	31.87 ± 2.99	0.27 ± 0.01	0.90 ± 0.04
DnaJ-A1-pTyr381ΔC	15.23 ± 1.26	2.75 ± 0.21	0.55 ± 0.05	12.29 ± 1.11	0.20 ± 0.01	1.12 ± 0.09
VCP-pTyr805-tail7	10.08 ± 1.20	2.20 ± 0.24	0.40 ± 0.05	10.10 ± 1.58	0.62 ± 0.11	0.74 ± 0.12
VCP-pTyr805-tail2	9.38 ± 1.00	0.99 ± 0.09	0.81 ± 0.08	N/A	N/A	0.12 ± 0.01

	L. dumoffii His-LeSH2	*L. anisa* LeSH2a	*L. anisa* LeSH2b	*L. pneumophila* LeSH2	*L. waltersii* LeSH5 tSH2	His-Src SH2 wt	His-Src SH2 TrM (superbinder)
Shc1-pTyr239	2.46 ± 0.19	3.05 ± 0.26	38.07 ± 10.45	3.66 ± 0.43	6.17 ± 0.84	1.07 ± 0.15	0.017 ± 0.003
Shc1-pTyr317	9.35 ± 1.78	0.94 ± 0.07	40.60 ± 5.63	8.77 ± 1.26	13.79 ± 1.48	2.81 ± 0.38	0.033 ± 0.007
EGFR-pTyr954	>50	7.98 ± 1.67	>50	44.62 ± 12.55	6.20 ± 0.48	13.26 ± 1.65	0.021 ± 0.002
EGFR-pTyr1086	14.70 ± 1.25	4.45 ± 0.57	>50	31.38 ± 5.65	33.52 ± 10.94	10.88 ± 2.23	0.122 ± 0.013
MidT-pTyr324	1.23 ± 0.26	0.42 ± 0.02	36.86 ± 11.10	1.32 ± 0.10	5.11 ± 0.62	0.27 ± 0.04	0.015 ± 0.005
GGpYGG	14.65 ± 1.82	1.38 ± 0.14	35.20 ± 6.56	9.82 ± 0.79	>50	>50	1.497 ± 0.136
IL2Rβ-pTyr387	2.94 ± 0.21	0.15 ± 0.01	4.00 ± 0.45	1.71 ± 0.10	18.00 ± 1.35	4.13 ± 0.31	0.402 ± 0.028
DnaJ-A1-pTyr381	1.12 ± 0.10	0.57 ± 0.18	18.68 ± 3.49	0.16 ± 0.01	29.18 ± 5.13	5.17 ± 0.57	0.223 ± 0.017
DnaJ-A1-pTyr381ΔN	0.71 ± 0.07	0.55 ± 0.03	22.59 ± 1.20	0.10 ± 0.01	14.98 ± 0.87	2.57 ± 0.13	0.114 ± 0.007
DnaJ-A1-pTyr381ΔC	5.26 ± 0.34	0.34 ± 0.03	16.51 ± 1.68	1.09 ± 0.06	28.66 ± 2.53	10.22 ± 0.91	0.407 ± 0.025
VCP-pTyr805-tail7	9.10 ± 1.77	2.53 ± 0.42	31.17 ± 11.12	0.81 ± 0.18	42.56 ± 7.02	9.77 ± 1.31	1.184 ± 0.199
VCP-pTyr805-tail2	7.43 ± 0.94	N/A	N/A	N/A	N/A	>50	1.786 ± 0.257

	L. dumoffii His-LeSH1a D44S	*L. pneumophila* His-RavO SH2 T310A	*L. longbeachae* His-LeSH P85A	*L. longbeachae* His-LeSH R46K
Shc1-pTyr239	>50	1.52 ± 0.12	14.95 ± 1.04	>50
Shc1-pTyr317	20.68 ± 2.65	2.68 ± 0.20	11.93 ± 0.72	>50
MidT-pTyr324	45.41 ± 9.41	11.23 ± 1.25	7.30 ± 0.67	>50
GGpYGG	>50	>50	11.51 ± 0.57	>50
IL2Rβ-pTyr387	21.13 ± 2.11	24.16 ± 2.36	3.69 ± 0.27	4.01 ± 0.30
DnaJ-A1-pTyr381	>50	46.08 ± 7.34	1.64 ± 0.17	3.03 ± 0.43
VCP-pTyr805-tail7	>50	17.43 ± 2.53	5.85 ± 0.76	43.22 ± 6.71
VCP-pTyr805-tail2	>50	20.98 ± 2.96	4.29 ± 0.55	>50

Supplementary Table 3. Peptide array results on Array-1, probed with either GST-LeSH or the GST-RavO SH2 domain

		Spot intensity				Relative SMALI score		
Spot	Sequence	LeSH	RavO SH2	Gene name	pTyr position	Fyn SH2	Grb2 SH2	Cysteine?
A1	EPQpYEEIPIYG	58	50	Hamster polyomavirus middle-T antigen	324	**1.40**	**1.05**	
A2	GGpYGGG	61	37	GGpYGG	designed	–	–	
A3	DHQpYYNDFPGG	43	63	Shc1 (*)	239	0.72	**1.13**	
A4	HQYpYNDFPGKG	26	27	Shc1 (*)	240	0.40	0.56	
A5	DPSpYVNVQNLG	35	68	Shc1 (*)	317	0.96	**1.47**	
A6	EPQ YEEIPIYG	18	1	non-phospho-A1	–	–	–	
A7	GG YGGG	19	1	non-phospho-A2	–	–	–	
A8	DHQ YYNDFPGG	19	1	non-phospho-A3	–	–	–	
A9	HQY YNDFPGKG	23	0	non-phospho-A4	–	–	–	
A10	DPS YVNVQNLG	18	1	non-phospho-A5	–	–	–	
A11	PQRpYLVIQGDG	52	53	EGFR (*)	954	0.71	0.62	
A12	SNFpYRALMDEG	52	46	EGFR (*)	974	0.87	0.66	
A13	ADEpYLIPQQGG	66	72	EGFR (*)	992	0.54	0.51	
A14	LQRpYSSDPTGG	45	60	EGFR (*)	1045	0.32	0.66	
A15	VPEpYINQSVPG	20	73	EGFR (*)	1068	0.60	**1.49**	
A16	NPVpYHNQPLNG	30	58	EGFR (*)	1086	0.53	**1.20**	
A17	DPHpYQDPHSTG	25	29	EGFR (*)	1101	0.62	0.63	
A18	NPEpYLNTVQPG	36	65	EGFR (*)	1114	0.58	**1.45**	
A19	NPDpYQQDFFPG	76	52	EGFR (*)	1148	0.50	0.67	
A20	NAEpYLRVAPQG	26	48	EGFR (*)	1173	0.60	0.83	
A21	APEpYENIRHYG	17	31	FGD6; KIAA1362; ZFYVE24.	748	**1.48**	**1.38**	
A22	IRHpYEEIPEYG	20	32	FGD6; KIAA1362; ZFYVE24.	754	**1.33**	0.91	
A23	FPIpYENVNPEG	19	56	PTPRB; PTPB.	1981	**1.31**	**1.54**	
A24	GEGpYEEPDSEG	27	47	CD19.	409	**1.25**	0.89	
A25	NPDpYEPIRKGG	24	22	CD3E; T3E.	188	**1.24**	0.71	
B1	EHPpYELLLTAG	81	52	ANKS1A; ANKS1; KIAA0229.	454	**1.16**	**1.07**	
B2	DEEpYEVPDLDG	49	63	PDE2A.	920	**1.15**	0.84	
B3	TKLpYDMIADLG	20	34	STRN3; GS2NA; SG2NA.	374	**1.13**	0.64	
B4	CPHpYEKVSGDG	22	39	EFNB2; EPLG5; HTKL; LERK5.	304	**1.11**	0.96	Yes
B5	CSMpYEDISRGG	75	60	CD79A; IGA; MB1.	199	**1.11**	0.88	Yes
B6	SYDpYDLIIIGG	47	51	TXNRD1.	13	**1.09**	0.58	
B7	TATpYEDIVTLG	74	65	CD79B; B29; IGB.	207	**1.08**	0.97	
B8	YAVpYETPTAHG	31	43	TOLLIP.	86	**1.06**	0.76	
B9	DGDpYEFLKSWG	31	20	STK4; MST1.	433	**1.04**	0.70	
B10	GHEpYTNIKYSG	30	18	PTPN11; PTP2C; SHPTP2.	546	**1.03**	**1.18**	
B11	NKTpYETVASLG	49	50	DDEFL1; UPLC1.	733	**1.02**	0.84	
B12	SQDpYDQLPSCG	42	50	CBLB; RNF56; Nbla00127.	889	**1.01**	0.55	Yes
B13	EIEpYENQKRLG	40	58	FLT3; STK1.	768	1.00	**1.25**	
B14	REApYEEPPEQG	71	63	GPA33.	281	0.99	0.90	
B15	PSEpYDLLWVPG	27	41	ALS2CR19; PAR3B; PAR3L.	1056	0.98	0.49	
B16	DEVpYDDVDTSG	52	61	FYB; SLAP130.	651	0.98	0.67	
B17	NRYpYDEDEDEG	63	42	LIMA1; EPLIN; SREBP3; PP624.	752	0.97	0.63	
B18	ITIpYSTINHSG	30	52	SLAMF6; KALI; UNQ6123/PRO20080.	308	0.97	0.71	
B19	EDDpYESPNDDG	48	44	LCP2.	113	0.96	0.55	
B20	KRPpYFTVDEAG	73	53	PVRL1; HVEC; PRR1.	468	0.95	0.65	
B21	RILpYQNLNEPG	35	49	AKAP9; AKAP350; AKAP450; KIAA0803.	3465	0.94	**1.38**	
B22	SQLpYTNPDSRG	50	45	DOCK4; KIAA0716.	821	0.94	**1.28**	
B23	APSpYLEISSMG	23	44	GTF2I; BAP135; WBSCR6.	920	0.94	0.93	
B24	ESPpYQELQGQG	26	52	TYROBP; DAP12; KARAP.	91	0.93	0.76	
B25	EPTpYLVIDPRG	42	51	ADCY4.	444	0.93	0.90	
C1	EASpYVNLPTIG	50	61	RPSA; LAMBR; LAMR1.	138	0.90	**1.55**	
C2	QAQpYDTPKAGG	23	31	DCBLD2; CLCP1; ESDN.	732	0.89	0.26	
C3	ADEpYDQPWEWG	23	36	SHB.	336	0.89	0.34	
C4	KDTpYDALHMQG	23	57	CD247; CD3Z; T3Z; TCRZ.	153	0.88	0.51	
C5	PFTpYEDPNQAG	77	69	EPHA4; HEK8; SEK.	602	0.88	0.69	
C6	NTDpYTELHQQG	98	64	GOLGA5; RETII; RFG5.	42	0.88	0.75	
C7	DDLpYDQDDSRG	93	63	LSR; LISCH.	535	0.88	0.57	
C8	FEGpYVELPPIG	95	65	CSF2RB; IL3RB; IL5RB.	766	0.87	**1.04**	
C9	TSVpYYTVTSGG	35	26	PPP1R16B; ANKRD4; KIAA0823.	536	0.87	0.59	
C10	MEDpYDYVHLQG	54	29	BCAR1; CAS; CRKAS.	664	0.86	0.53	
C11	PTEpYASICVRG	27	21	BTLA.	282	0.86	0.76	Yes
C12	GHEpYIYVDPMG	40	49	PDGFRB.	579	0.86	**1.01**	
C13	KFHpYDNTAGIG	52	55	KDR; FLK1.	1214	0.86	**1.09**	
C14	TPGpYVAPEVLG	28	72	CAMK1.	184	0.86	0.86	
C15	QGFpYVALRLVG	33	43	EPS15L1; EPS15R.	74	0.85	0.75	
C16	EVTpYAQLDHWG	46	40	LAIR1; CD305.	251	0.84	0.96	
C17	GLEpYSGIQELG	47	52	PKN2; PRK2; PRKCL2.	635	0.84	0.70	
C18	QGDpYINLQTKG	25	64	RAB7L1.	190	0.84	**1.32**	
C19	EIIpYSEVKKQG	25	34	CEACAM1; BGP; BGP1.	520	0.84	0.78	
C20	DNIpYEWRSTIG	31	25	UBE2E3; UBCE4; UBCH9.	91	0.84	0.75	
C21	DYDpYVHLQGKG	24	36	NEDD9; CASL.	631	0.83	0.72	
C22	KRSpYEEHIPYG	32	30	INSR.	1355	0.83	0.77	
C23	AKApYDHLFKLG	17	21	RAB13.	5	0.83	0.52	
C24	GKDpYDPVAARG	20	30	MAPRE1.	123	0.83	0.52	
C25	PDTpYEDPSLAG	61	63	EPHA6; EHK2.	611	0.82	0.73	
D1	NPVpYATLYMGG	34	19	LRP1; A2MR.	4507	0.80	0.77	
D2	ISRpYETSSTSG	33	50	PPP1R12A; MBS; MYPT1.	890	0.80	0.73	
D3	SFGpYDKPHVLG	23	47	IFNGR1.	457	0.80	0.40	
D4	NSDpYCGISEGG	32	46	CASP8AP2; FLASH; KIAA1315; RIP25.	739	0.79	0.61	Yes
D5	IESpYQNLTRVG	72	54	ABI1; SSH3BP1.	22	0.79	**1.38**	
D6	DKEpYYSVHNKG	19	16	MET.	1234	0.79	0.55	
D7	DGTpYETQGGKG	28	18	EPHA1; EPH; EPHT; EPHT1.	781	0.79	0.80	
D8	RGLpYDGPVCEG	37	42	DPYSL2; CRMP2.	499	0.79	0.60	Yes
D9	DPIpYEDRVYQG	57	35	CTNND2; NPRAP.	424	0.78	0.83	
D10	PHMpYEDAQLQG	81	41	RGS17.	171	0.78	0.76	
D11	VGPpYELGMEHG	17	27	RPS6KB1.	62	0.78	0.93	
D12	KSRpYSDLDFEG	80	53	BAI3; KIAA0550.	1419	0.78	0.73	
D13	MEPpYEAQRIMG	67	48	SHB.	272	0.78	0.67	
D14	MIIpYSCLKEEG	21	38	STAT1.	106	0.78	0.70	Yes
D15	NVVpYSEVRIIG	50	46	FCRL5; FCRH5; IRTA2; UNQ503/PRO820.	924	0.77	0.84	
D16	ETVpYSEVRKAG	35	35	PECAM1.	713	0.77	0.77	
D17	CPDpYDLCSVCG	14	20	SQSTM1; ORCA.	148	0.76	0.54	Yes
D18	TPGpYQAPEIRG	39	55	LRRK1; KIAA1790.	1453	0.76	0.72	
D19	AGHpYEDTILKG	28	45	ALK.	1604	0.76	0.85	
D20	GEApYEDDEHHG	48	47	DNAJA1; DNAJ2; HDJ2; HSJ2; HSPF4.	381	0.75	0.88	
D21	LDGpYDRDDKEG	59	53	ANP32A; C15orf1; LANP; MAPM; PHAP1.	148	0.75	0.51	
D22	LGVpYSYIPLVG	19	37	PTPN22; PTPN8.	526	0.75	0.85	
D23	EDIpYESRHEIG	56	56	FRK.	387	0.75	0.59	
D24	ATLpYAVVENVG	21	57	TNFRSF1A; TNFAR; TNFR1.	360	0.74	0.93	
D25	PQApYFTLPRNG	34	61	DVL2.	362	0.74	0.64	

(*) Residue numbers of Shc1 and EGFR follow the convention (van der Geer *et al.* 1996, *Curr Biol.*, 6(11):1435-44, Foley *et al*., 2010, *Semin Cell Dev Biol.*, 21(9):951-60).
The SMALI score > 1 is in bold.

Supplementary Table 4. Peptide array results on Array-2, probed with either GST-LeSH or the GST-RavO SH2 domain

		Spot intensity				Relative SMALI score		
Spot	Sequence	LeSH	RavO SH2	Gene name	pTyr position	Fyn SH2	Grb2 SH2	Cysteine?
E1	EPQpYEEIPIYG	57	63	Hamster polyomavirus middle-T antigen	324	**1.40**	**1.05**	
E2	GGpYGGG	60	34	GGpYGGG	designed	–	–	
E3	DHQpYYNDFPGG	49	85	Shc1 (*)	239	0.72	**1.13**	
E4	HQYpYNDFPGKG	26	26	Shc1 (*)	240	0.40	0.56	
E5	DPSpYVNVQNLG	28	92	Shc1 (*)	317	0.96	**1.47**	
E6	EPQ YEEIPIYG	6	5	non-phospho-A1	–	–	–	
E7	GG YGGG	9	4	non-phospho-A2	–	–	–	
E8	DHQ YYNDFPGG	12	5	non-phospho-A3	–	–	–	
E9	HQY YNDFPGKG	18	4	non-phospho-A4	–	–	–	
E10	DPS YVNVQNLG	10	4	non-phospho-A5	–	–	–	
E11	PQRpYLVIQGDG	41	42	EGFR (*)	954	0.71	0.62	
E12	SNFpYRALMDEG	58	16	EGFR (*)	974	0.87	0.66	
E13	ADEpYLIPQQGG	37	67	EGFR (*)	992	0.54	0.51	
E14	LQRpYSSDPTGG	47	22	EGFR (*)	1045	0.32	0.66	
E15	VPEpYINQSVPG	24	72	EGFR (*)	1068	0.60	**1.49**	
E16	NPVpYHNQPLNG	37	65	EGFR (*)	1086	0.53	**1.20**	
E17	DPHpYQDPHSTG	24	21	EGFR (*)	1101	0.62	0.63	
E18	NPEpYLNTVQPG	24	90	EGFR (*)	1114	0.58	**1.45**	
E19	NPDpYQQDFFPG	70	62	EGFR (*)	1148	0.50	0.67	
E20	NAEpYLRVAPQG	72	65	EGFR (*)	1173	0.60	0.83	
E21	EILpYVNMDEGG	64	93	AXL; UFO.	814	0.86	**1.67**	
E22	SNPpYENSLIPG	61	65	EPOR.	489	**1.06**	**1.58**	
E23	ADSpYENMDNPG	56	32	CD19.	531	**1.01**	**1.53**	
E24	SREpYVNVSQEG	72	92	LAT.	220	0.89	**1.51**	
E25	DKVpYENVTGLG	18	25	PTK2; FAK; FAK1.	925	**1.13**	**1.47**	
F1	AALpYKNLLHSG	16	10	KIT.	703	0.83	**1.37**	
F2	SADpYMNLHFKG	29	25	UNC13B; UNC13.	1033	0.83	**1.35**	
F3	QNGpYENPTYKG	30	17	APP; A4; AD1.	757	**1.22**	**1.33**	
F4	DPYpYGNDSDFG	62	43	ACP1.	132	0.65	**1.30**	
F5	SQLpYTNPDSRG	64	44	DOCK4; KIAA0716.	821	0.94	**1.28**	
F6	DSVpYANWMLSG	49	16	RET.	1096	0.46	**1.27**	
F7	DQApYANSQPAG	36	20	SIT1; SIT.	188	0.47	**1.25**	
F8	SSDpYINANYIG	59	81	PTPRK; PTPK.	940	0.51	**1.24**	
F9	ERDpYTNLPSSG	84	64	CSF1R; FMS.	923	0.81	**1.23**	
F10	AMFpYTNRVLKG	49	26	CAP1; CAP.	163	0.56	**1.22**	
F11	EDMpYTNGSPAG	47	31	MPP1; DXS552E; EMP55.	48	0.41	**1.21**	
F12	MAPpYDNYVPSG	30	27	PDGFRB.	775	0.82	**1.19**	
F13	DSDpYENTQSGG	55	33	GIT1.	598	0.98	**1.16**	
F14	EDCpYGNYDNLG	66	57	PDPK1; PDK1.	373	0.59	**1.16**	Yes
F15	SNIpYVEVEDEG	83	93	SH2D2A; SCAP; TSAD; VRAP.	305	**1.11**	**1.12**	
F16	RDIpYKNPDYVG	18	34	FLT1; FLT; FRT.	1048	0.85	**1.11**	
F17	AMEpYYNWGRFG	24	37	LAT2; LAB; NTAL; WBS15; WBSCR15; WBSCR5; HSPC046.	118	0.70	**1.10**	
F18	NLPpYVQILKTG	38	79	FGFR1; FGFBR; FLG; FLT2.	307	0.95	**1.08**	
F19	GPVpYIGELPQG	54	79	TOLLIP.	13	0.32	**1.08**	
F20	SEVpYEIMVKCG	53	42	PDGFRA.	926	0.64	**1.06**	Yes
F21	QGSpYVPLLRDG	94	100	LSR; LISCH.	372	0.68	**1.05**	
F22	CVVpYEDMSHSG	91	69	CD7.	222	0.70	**1.04**	Yes
F23	NPDpYWNHSLPG	39	52	ERBB4; HER4.	1242	0.55	**1.03**	
F24	PHTpYQNRRPFG	65	33	FLT3; STK1.	969	0.46	**1.03**	
F25	PLVpYVIVGKRG	9	21	BDKRB2; BKR2.	332	0.66	**1.02**	
G1	HNEpYVRDLPVG	86	78	UNC13B; UNC13.	1047	0.56	0.98	
G2	IDVpYMIMVKCG	38	17	EGFR(*)	920	0.20	0.97	Yes
G3	NLEpYVSVSPTG	80	74	SLAMF6; KALI; UNQ6123/PRO20080.	273	0.75	0.96	
G4	GSSpYEEEEEG	81	30	PVRL1; HVEC; PRR1.	436	0.99	0.96	
G5	ENPpYSEVGKIG	60	13	SHANK2; KIAA1022.	393	0.95	0.96	
G6	AELpYEKLPQGG	23	7	TEK; TIE2.	1048	**1.04**	0.96	
G7	SQApYEVLSDAG	52	36	DNAJA1; DNAJ2; HDJ2; HSJ2; HSPF4.	52	**1.02**	0.95	
G8	NLSpYTEILKIG	76	84	ELMO1; KIAA0281.	511	0.87	0.95	
G9	SVMpYTVVPQMG	44	45	OR7G1.	278	0.57	0.95	
G10	TPIpYLDILGG	81	86	NTRK3; TRKC.	834	0.78	0.94	
G11	ATLpYAVVENVG	41	41	TNFRSF1A; TNFAR; TNFR1.	360	0.74	0.93	
G12	RPDpYIIVTQRG	76	85	AHR.	378	0.66	0.93	
G13	SADpYVLSVRDG	88	95	PTK6; BRK.	114	0.43	0.93	
G14	YDPpYSEEDPDG	78	47	IL2RB.	387	0.73	0.93	
G15	EPVpYSMEAADG	55	51	CTTN; EMS1.	446	0.50	0.93	
G16	TAApYQELCRQG	58	20	PTPRN2.	666	0.80	0.92	Yes
G17	PGPpYVGQAGTG	11	58	CTNND1; KIAA0384.	193	0.45	0.92	
G18	QDYpYEVVPPNG	62	67	PLEKHB1; KPL1; PHR1.	176	**1.05**	0.92	
G19	SVFpYAIVTPMG	56	63	OR2D2; OR2D1.	276	0.69	0.91	
G20	AEApYSEIGMKG	25	15	CD247; CD3Z; T3Z; TCRZ.	123	0.87	0.91	
G21	DLQpYITVSKEG	53	96	MLLT4; AF6.	1480	0.63	0.91	
G22	DGGpYMDMSKDG	33	17	PDGFRB.	740	0.22	0.90	
G23	DVVpYLKVAKPG	0	5	DLG4; PSD95.	240	0.54	0.90	
G24	TRSpYVILSFEG	57	27	PDGFRA.	720	0.69	0.90	
G25	FREpYEYDLKWG	43	9	FLT3; STK1.	597	0.77	0.90	
H1	RFIpYEFEHFNG	100	95	PPP2R5B.	244	0.78	0.88	
H2	PGLpYSKTMTPG	23	7	PLEKHC1; KIND2; MIG2.	185	0.40	0.88	
H3	VNGpYVMPDTHG	45	84	ERBB3; HER3.	1159	0.99	0.88	
H4	GEApYEDDEHHG	74	42	DNAJA1; DNAJ2; HDJ2; HSJ2; HSPF4.	381	0.75	0.88	
H5	DGLpYQGLSTAG	38	24	CD247; CD3Z; T3Z; TCRZ.	142	0.67	0.87	
H6	RLIpYEDYVSIG	72	80	RGS19; GAIP; GNAI3IP.	143	0.68	0.87	
H7	HRIpYSYVVSRG	40	12	UBXD8; ETEA; KIAA0887.	79	0.59	0.87	
H8	EVEpYSTVASPG	36	10	CD300A; CMRF35H.	255	0.81	0.87	
H9	SDPpYAIVSFLG	76	20	DYSF; FER1L1.	1176	0.59	0.87	
H10	AKLpYSLVIWGG	15	5	BDKRB2; BKR2.	177	0.60	0.87	
H11	DDQpYVSSVGTG	59	35	BMX.	566	0.39	0.87	
H12	VNNpYSEAEIKG	81	18	EPN2; KIAA1065.	17	0.77	0.86	
H13	EEVpYVKHMGNG	14	5	FLNC; ABPL; FLN2.	2683	0.38	0.86	
H14	KDIpYTELSIAG	82	26	MRIP; KIAA0864; RHOIP3.	944	0.84	0.85	
H15	TFDpYILCMDEG	75	85	ACP1.	87	0.41	0.85	Yes
H16	KLLpYAKDIPTG	9	9	PLXNC1; VESPR.	1471	0.32	0.85	
H17	NVSpYEHSFNKG	30	43	GPR126.	1196	0.73	0.85	
H18	EELpYSKVTPRG	35	10	STS1; KIAA1959.	19	0.62	0.85	
H19	GEVpYEGVWKKG	4	5	ABL1; ABL; JTK7.	257	0.93	0.85	
H20	EGDpYMVLPRRG	70	31	BAI2.	1339	0.61	0.85	
H21	QPCpYETINRIG	79	71	GDI2; RABGDIB.	203	**1.39**	0.84	Yes
H22	NKTpYETVASLG	75	31	DDEFL1; UPLC1.	733	**1.02**	0.84	
H23	SITpYAAVARHG	74	20	LAIR1; CD305.	281	0.59	0.84	
H24	SKYpYTPVLAKG	9	2	STAT5A; STAT5.	683	0.62	0.84	
H25	KIRpYESLTDPG	60	24	HSP90AB1; HSP90B; HSPC2; HSPCB.	55	**1.12**	0.84	

(*) Residue numbers of Shc1 and EGFR follow the convention (van der Geer *et al.* 1996, *Curr Biol.*, 6(11):1435-44, Foley *et al*., 2010, *Semin Cell Dev Biol.*, 21(9):951-60).
The SMALI score > 1 is in bold.

Supplementary Table 5. Z-scores from spot intensities probed with GST-LeSH. The sequences are sorted by the Z-score. Z-scores above 0.6 are in bold. The intensities are averaged for the 24 pairs of replicate peptides in the two membranes. Non-phosphorylated peptides are excluded.

Spot (non-redundant, Cys-less)	Peptide sequence	Intensity	Z-score
H1	RFIpYEFEHFNG	100	**2.368**
C6	NTDpYTELHQQG	98	**2.281**
C8	FEGpYVELPPIG	95	**2.149**
F21	QGSpYVPLLRDG	94	**2.105**
C7	DDLpYDQDDSRG	93	**2.061**
G13	SADpYVLSVRDG	88	**1.842**
G1	HNEpYVRDLPVG	86	**1.754**
F9	ERDpYTNLPSSG	84	**1.667**
F15	SNIpYVEVEDEG	83	**1.623**
H14	KDIpYTELSIAG	82	**1.579**
B1	EHPpYELLLTAG	81	**1.535**
D10	PHMpYEDAQLQG	81	**1.535**
G4	GSSpYEEEEEEG	81	**1.535**
G10	TPIpYLDILGG	81	**1.535**
H12	VNNpYSEAEIKG	81	**1.535**
D12	KSRpYSDLDFEG	80	**1.491**
G3	NLEpYVSVSPTG	80	**1.491**
G14	YDPpYSEEDPDG	78	**1.404**
C5	PFTpYEDPNQAG	77	**1.360**
G8	NLSpYTEILKIG	76	**1.316**
G12	RPDpYIIVTQRG	76	**1.316**
H9	SDPpYAIVSFLG	76	**1.316**
B7	TATpYEDIVTLG	74	**1.228**
H23	SITpYAAVARHG	74	**1.228**
<A19 = E19>	NPDpYQQDFFPG	73	**1.184**
B20	KRPpYFTVDEAG	73	**1.184**
D5	IESpYQNLTRVG	72	**1.140**
E24	SREpYVNVSQEG	72	**1.140**
H6	RLIpYEDYVSIG	72	**1.140**
B14	REApYEEPPEQG	71	**1.096**
H20	EGDpYMVLPRRG	70	**1.053**
D13	MEPpYEAQRIMG	67	**0.921**
F24	PHTpYQNRRPFG	65	**0.833**
E21	EILpYVNMDEGG	64	**0.789**
B17	NRYpYDEDEDEG	63	**0.746**
<B11 = H22>	NKTpYETVASLG	62	**0.702**
F4	DPYpYGNDSDFG	62	**0.702**
G18	QDYpYEVVPPNG	62	**0.702**
<D20 = H4>	GEApYEDDEHHG	61	**0.658**
C25	PDTpYEDPSLAG	61	**0.658**
E22	SNPpYENSLIPG	61	**0.658**
<A2 = E2>	GGpYGGG	60	**0.614**
G5	ENPpYSEVGKIG	60	**0.614**
H25	KIRpYESLTDPG	60	**0.614**
D21	LDGpYDRDDKEG	59	0.570
F8	SSDpYINANYIG	59	0.570
H11	DDQpYVSSVGTG	59	0.570
<A1 = E1>	EPQpYEEIPIYG	58	0.526
<B22 = F5>	SQLpYTNPDSRG	57	0.482
D9	DPIpYEDRVYQG	57	0.482
G24	TRSpYVILSFEG	57	0.482
D23	EDIpYESRHEIG	56	0.439
E23	ADSpYENMDNPG	56	0.439
G19	SVFpYAIVTPMG	56	0.439
<A12 = E12>	SNFpYRALMDEG	55	0.395
F13	DSDpYENTQSGG	55	0.395
G15	EPVpYSMEAADG	55	0.395
C10	MEDpYDYVHLQG	54	0.351
F19	GPVpYIGELPQG	54	0.351
G21	DLQpYITVSKEG	53	0.307
B16	DEVpYDDVDTSG	52	0.263
C13	KFHpYDNTAGIG	52	0.263
G7	SQApYEVLSDAG	52	0.263
<A13 = E13>	ADEpYLIPQQGG	51	0.219
C1	EASpYVNLPTIG	50	0.175
D15	NVVpYSEVRIIG	50	0.175
<A20 = E20>	NAEpYLRVAPQG	49	0.132
B2	DEEpYEVPDLDG	49	0.132
F6	DSVpYANWMLSG	49	0.132
F10	AMFpYTNRVLKG	49	0.132
B19	EDDpYESPNDDG	48	0.088
<A11 = E11>	PQRpYLVIQGDG	47	0.044
B6	SYDpYDLIIIGG	47	0.044
C17	GLEpYSGIQELG	47	0.044
F11	EDMpYTNGSPAG	47	0.044
<A3 = E3>	DHQpYYNDFPGG	46	0.000
<A14 = E14>	LQRpYSSDPTGG	46	0.000
C16	EVTpYAQLDHWG	46	0.000
H3	VNGpYVMPDTHG	45	-0.044
G9	SVMpYTVVPQMG	44	-0.088
G25	FREpYEYDLKWG	43	-0.132
B25	EPTpYLVIDPRG	42	-0.175
B13	EIEpYENQKRLG	40	-0.263
C12	GHEpYIYVDPMG	40	-0.263
H7	HRIpYSYVVSRG	40	-0.263
D18	TPGpYQAPEIRG	39	-0.307
F23	NPDpYWNHSLPG	39	-0.307
F18	NLPpYVQILKTG	38	-0.351
H5	DGLpYQGLSTAG	38	-0.351
F7	DQApYANSQPAG	36	-0.439
H8	EVEpYSTVASPG	36	-0.439
B21	RILpYQNLNEPG	35	-0.482
C9	TSVpYYTVTSGG	35	-0.482
D16	ETVpYSEVRKAG	35	-0.482
H18	EELpYSKVTPRG	35	-0.482
D1	NPVpYATLYMGG	34	-0.526
D25	PQApYFTLPRNG	34	-0.526
<A16 = E16>	NPVpYHNQPLNG	33	-0.570
C15	QGFpYVALRLVG	33	-0.570
D2	ISRpYETSSTSG	33	-0.570
G22	DGGpYMDMSKDG	33	-0.570
C22	KRSpYEEHIPYG	32	-0.614
<A5 = E5>	DPSpYVNVQNLG	31	-0.658
<D24 = G11>	ATLpYAVVENVG	31	-0.658
B8	YAVpYETPTAHG	31	-0.658
B9	DGDpYEFLKSWG	31	-0.658
C20	DNIpYEWRSTIG	31	-0.658
<A18 = E18>	NPEpYLNTVQPG	30	-0.702
B10	GHEpYTNIKYSG	30	-0.702
B18	ITIpYSTINHSG	30	-0.702
F3	QNGpYENPTYKG	30	-0.702
F12	MAPpYDNYVPSG	30	-0.702
H17	NVSpYEHSFNKG	30	-0.702
F2	SADpYMNLHFKG	29	-0.746
C14	TPGpYVAPEVLG	28	-0.789
D7	DGTpYETQGGKG	28	-0.789
D19	AGHpYEDTILKG	28	-0.789
A24	GEGpYEEPDSEG	27	-0.833
B15	PSEpYDLLWVPG	27	-0.833
<A4 = E4>	HQYpYNDFPGKG	26	-0.877
B24	ESPpYQELQGQG	26	-0.877
C18	QGDpYINLQTKG	25	-0.921
C19	EIIpYSEVKKQG	25	-0.921
G20	AEApYSEIGMKG	25	-0.921
<A17 = E17>	DPHpYQDPHSTG	24	-0.965
A25	NPDpYEPIRKGG	24	-0.965
C21	DYDpYVHLQGKG	24	-0.965
F17	AMEpYYNWGRFG	24	-0.965
B23	APSpYLEISSMG	23	-1.009
C2	QAQpYDTPKAGG	23	-1.009
C3	ADEpYDQPWEWG	23	-1.009
C4	KDTpYDALHMQG	23	-1.009
D3	SFGpYDKPHVLG	23	-1.009
G6	AELpYEKLPQGG	23	-1.009
H2	PGLpYSKTMTPG	23	-1.009
<A15 = E15>	VPEpYINQSVPG	22	-1.053
A22	IRHpYEEIPEYG	20	-1.140
B3	TKLpYDMIADLG	20	-1.140
C24	GKDpYDPVAARG	20	-1.140
A23	FPIpYENVNPEG	19	-1.184
D6	DKEpYYSVHNKG	19	-1.184
D22	LGVpYSYIPLVG	19	-1.184
E25	DKVpYENVTGLG	18	-1.228
F16	RDIpYKNPDYVG	18	-1.228
A21	APEpYENIRHYG	17	-1.272
C23	AKApYDHLFKLG	17	-1.272
D11	VGPpYELGMEHG	17	-1.272
F1	AALpYKNLLHSG	16	-1.316
H10	AKLpYSLVIWGG	15	-1.360
H13	EEVpYVKHMGNG	14	-1.404
G17	PGPpYVGQAGTG	11	-1.535
F25	PLVpYVIVGKRG	9	-1.623
H16	KLLpYAKDIPTG	9	-1.623
H24	SKYpYTPVLAKG	9	-1.623
H19	GEVpYEGVWKKG	4	-1.842
G23	DVVpYLKVAKPG	0	-2.018

Supplementary Table 6. Z-scores from spot intensities probed with GST-RavO SH2. The sequences are sorted by the Z-score. Z-scores above 0.6 are in bold. The intensities are averaged for the 24 pairs of replicate peptides in the two membranes. Non-phosphorylated peptides are excluded.

Spot (non-redundant, Cys-less)	Peptide sequence	Intensity	Z-score	Spot (non-redundant, Cys-less)	Peptide sequence	Intensity	Z-score
F21	QGSpYVPLLRDG	100	**2.414**	F4	DPYpYGNDSDFG	43	-0.037
G21	DLQpYITVSKEG	96	**2.256**	C15	QGFpYVALRLVG	43	-0.047
H1	RFIpYEFEHFNG	95	**2.212**	H17	NVSpYEHSFNKG	43	-0.051
G13	SADpYVLSVRDG	95	**2.194**	B8	YAVpYETPTAHG	43	-0.053
E21	EILpYVNMDEGG	93	**2.111**	B17	NRYpYDEDEDEG	42	-0.068
F15	SNIpYVEVEDEG	93	**2.094**	B15	PSEpYDLLWVPG	41	-0.122
E24	SREpYVNVSQEG	92	**2.053**	<A14 = E14>	LQRpYSSDPTGG	41	-0.124
G10	TPIpYLDILGG	86	**1.831**	D10	PHMpYEDAQLQG	41	-0.144
G12	RPDpYIIVTQRG	85	**1.772**	<B11 = H22>	NKTpYETVASLG	41	-0.150
H3	VNGpYVMPDTHG	84	**1.736**	C16	EVTpYAQLDHWG	40	-0.189
G8	NLSpYTEILKIG	84	**1.732**	F17	AMEpYYNWGRFG	37	-0.312
F8	SSDpYINANYIG	81	**1.605**	D22	LGVpYSYIPLVG	37	-0.322
H6	RLIpYEDYVSIG	80	**1.560**	C3	ADEpYDQPWEWG	36	-0.345
<A5 = E5>	DPSpYVNVQNLG	80	**1.537**	C21	DYDpYVHLQGKG	36	-0.354
F19	GPVpYIGELPQG	79	**1.526**	G7	SQApYEVLSDAG	36	-0.365
F18	NLPpYVQILKTG	79	**1.510**	<A2 = E2>	GGpYGGG	35	-0.370
G1	HNEpYVRDLPVG	78	**1.477**	D9	DPIpYEDRVYQG	35	-0.372
<A18 = E18>	NPEpYLNTVQPG	77	**1.428**	H11	DDQpYVSSVGTG	35	-0.376
<A3 = E3>	DHQpYYNDFPGG	74	**1.301**	D16	ETVpYSEVRKAG	35	-0.404
G3	NLEpYVSVSPTG	74	**1.277**	C19	EIIpYSEVKKQG	34	-0.410
<A15 = E15>	VPEpYINQSVPG	73	**1.234**	F16	RDIpYKNPDYVG	34	-0.415
C14	TPGpYVAPEVLG	72	**1.222**	B3	TKLpYDMIADLG	34	-0.425
<A13 = E13>	ADEpYLIPQQGG	70	**1.105**	F24	PHTpYQNRRPFG	33	-0.456
C5	PFTpYEDPNQAG	69	**1.061**	F13	DSDpYENTQSGG	33	-0.470
G18	QDYpYEVVPPNG	67	**0.992**	E23	ADSpYENMDNPG	32	-0.501
C8	FEGpYVELPPIG	65	**0.920**	A22	IRHpYEEIPEYG	32	-0.534
E22	SNPpYENSLIPG	65	**0.905**	C2	QAQpYDTPKAGG	31	-0.540
B7	TATpYEDIVTLG	65	**0.894**	H20	EGDpYMVLPRRG	31	-0.564
C18	QGDpYINLQTKG	64	**0.858**	<A12 = E12>	SNFpYRALMDEG	31	-0.565
F9	ERDpYTNLPSSG	64	**0.848**	A21	APEpYENIRHYG	31	-0.567
C6	NTDpYTELHQQG	64	**0.847**	F11	EDMpYTNGSPAG	31	-0.570
B14	REApYEEPPEQG	63	**0.839**	G4	GSSpYEEEEEEG	30	-0.582
C25	PDTpYEDPSLAG	63	**0.826**	C24	GKDpYDPVAARG	30	-0.607
B2	DEEpYEVPDLDG	63	**0.823**	C22	KRSpYEEHIPYG	30	-0.623
G19	SVFpYAIVTPMG	63	**0.817**	C10	MEDpYDYVHLQG	29	-0.638
C7	DDLpYDQDDSRG	63	**0.798**	F12	MAPpYDNYVPSG	27	-0.728
<A16 = E16>	NPVpYHNQPLNG	61	**0.753**	D11	VGPpYELGMEHG	27	-0.740
B16	DEVpYDDVDTSG	61	**0.738**	<A4 = E4>	HQYpYNDFPGKG	27	-0.745
D25	PQApYFTLPRNG	61	**0.729**	G24	TRSpYVILSFEG	27	-0.752
C1	EASpYVNLPTIG	61	**0.713**	C9	TSVpYYTVTSGG	26	-0.756
B13	EIEpYENQKRLG	58	**0.623**	H14	KDIpYTELSIAG	26	-0.779
G17	PGPpYVGQAGTG	58	**0.607**	F10	AMFpYTNRVLKG	26	-0.792
C4	KDTpYDALHMQG	57	0.576	E25	DKVpYENVTGLG	25	-0.807
<A19 = E19>	NPDpYQQDFFPG	57	0.554	<A17 = E17>	DPHpYQDPHSTG	25	-0.816
<A1 = E1>	EPQpYEEIPIYG	57	0.543	C20	DNIpYEWRSTIG	25	-0.820
<A20 = E20>	NAEpYLRVAPQG	57	0.539	F2	SADpYMNLHFKG	25	-0.823
A23	FPIpYENVNPEG	56	0.512	H5	DGLpYQGLSTAG	24	-0.854
D23	EDIpYESRHEIG	56	0.502	H25	KIRpYESLTDPG	24	-0.876
D18	TPGpYQAPEIRG	55	0.487	A25	NPDpYEPIRKGG	22	-0.933
C13	KFHpYDNTAGIG	55	0.455	F25	PLVpYVIVGKRG	21	-0.987
D5	IESpYQNLTRVG	54	0.442	C23	AKApYDHLFKLG	21	-1.003
D12	KSRpYSDLDFEG	53	0.400	F7	DQApYANSQPAG	20	-1.020
D21	LDGpYDRDDKEG	53	0.381	B9	DGDpYEFLKSWG	20	-1.030
B20	KRPpYFTVDEAG	53	0.369	H9	SDPpYAIVSFLG	20	-1.031
B24	ESPpYQELQGQG	52	0.352	H23	SITpYAAVARHG	20	-1.036
B1	EHPpYELLLTAG	52	0.352	D1	NPVpYATLYMGG	19	-1.073
B18	ITIpYSTINHSG	52	0.333	H12	VNNpYSEAEIKG	18	-1.110
F23	NPDpYWNHSLPG	52	0.332	B10	GHEpYTNIKYSG	18	-1.115
C17	GLEpYSGIQELG	52	0.330	D7	DGTpYETQGGKG	18	-1.117
B25	EPTpYLVIDPRG	51	0.321	G22	DGGpYMDMSKDG	17	-1.151
B6	SYDpYDLIIIGG	51	0.313	F3	QNGpYENPTYKG	17	-1.183
G15	EPVpYSMEAADG	51	0.292	D6	DKEpYYSVHNKG	16	-1.190
D2	ISRpYETSSTSG	50	0.240	F6	DSVpYANWMLSG	16	-1.224
B21	RILpYQNLNEPG	49	0.236	G20	AEApYSEIGMKG	15	-1.242
C12	GHEpYIYVDPMG	49	0.226	G5	ENPpYSEVGKIG	13	-1.332
<D24 = G11>	ATLpYAVVENVG	49	0.201	H7	HRIpYSYVVSRG	12	-1.398
D13	MEPpYEAQRIMG	48	0.180	H18	EELpYSKVTPRG	10	-1.446
<A11 = E11>	PQRpYLVIQGDG	48	0.155	H8	EVEpYSTVASPG	10	-1.460
D3	SFGpYDKPHVLG	47	0.144	F1	AALpYKNLLHSG	10	-1.469
G14	YDPpYSEEDPDG	47	0.140	H16	KLLpYAKDIPTG	9	-1.505
A24	GEGpYEEPDSEG	47	0.120	G25	FREpYEYDLKWG	9	-1.528
D15	NVVpYSEVRIIG	46	0.101	H2	PGLpYSKTMTPG	7	-1.577
<D20 = H4>	GEApYEDDEHHG	45	0.038	G6	AELpYEKLPQGG	7	-1.610
D19	AGHpYEDTILKG	45	0.035	G23	DVVpYLKVAKPG	5	-1.690
G9	SVMpYTVVPQMG	45	0.025	H10	AKLpYSLVIWGG	5	-1.697
<B22 = F5>	SQLpYTNPDSRG	45	0.022	H13	EEVpYVKHMGNG	5	-1.702
B23	APSpYLEISSMG	44	0.018	H19	GEVpYEGVWKKG	5	-1.702
B19	EDDpYESPNDDG	44	-0.011	H24	SKYpYTPVLAKG	2	-1.810

Supplementary table 7. **Crystallographic data collection and refinement statistics**.

Crystal	apo LeSH	LeSH-phosphotyrosine complex	LeSH-IL2Rβ pTyr387 peptide complex	LeSH-DnaJA1 pTyr381 peptide complex	apo RavO SH2 domain	RavO SH2-Shc1 pTyr317 peptide complex
PDB code	6E8H	6E8I	6E8K	6E8M	6DM3	6DM4
**Data collection**						
Space group	$P2_12_12_1$	$P2_12_12_1$	$P2_12_12_1$	$P2_12_12_1$	$C2$	$P1$
Cell dimensions						
a, *b*, *c* (Å)	42.3, 66.5, 68.2	40.8, 64.1, 67.9	40.5, 64.2, 67.5	40.9, 64.7, 67.9	50.0, 73.0, 72.0	44.45, 45.04, 74.75
α, β, γ (º)	90, 90, 90	90, 90, 90	90, 90, 90	90, 90, 90	90, 109.9, 90	72.60, 90.14, 77.55
Resolution (Å)	19.6-1.66 (1.76-1.66)*	1.67-34.97 (1.76-1.67)	34.7-1.70 (1.79-1.70)	33.9-1.60 (1.69-1.60)	25.00-1.95 (1.98-1.95)	15.00 – 1.90 (1.93-1.90)
R_{merge}	0.052 (0.284)	0.058 (0.166)	0.052 (0.491)	0.052 (0.503)	0.058 (0.414)	0.050 (0.572)
Mean(I / σI)	68.2 (16.9)	8.5 (4.0)	12.6 (2.4)	24.9 (4.1)	28.6 (2.9)	33.19 (3.2)
Anomalous completeness (%)	97.5 (84.0)	85.4 (77.2)	91.3 (77.8)	90.3 (90.2)	N/A	N/A
Anomalous redundancy	29.4 (26.2)	1.7 (1.7)	2.8 (2.7)	5.8 (5.3)	N/A	N/A
**Refinement**						
Resolution (Å)	19.6-1.68	30.7-1.68	25.9-1.71	24.2-1.61	24.8-1.95	14.8-1.90
No. of reflections	42244	34765	34992	41196	16361	41046
R_{work} / R_{free}	0.212 / 0.248	0.217 / 0.250	0.188 / 0.211	0.199 / 0.212	0.261 / 0.318	0.183 / 0.230
Number of atoms						
SH2	1310	1302	1302	1302	1755	3883
Peptide	0	17 (pTyr)	29	21	0	171
ion	1 (chloride ion)	0	0	0	0	30 (sulfate ions)
Water	243	233	148	146	66	448
B-factors						
SH2	28.6	20.9	34.7	32.3	57.6	49.9
Peptide	n/a	16.3 (pTyr)	41.3	27.9	n/a	81.9
ion	36.8	n/a	n/a	n/a	n/a	75.6
Water	36.2	29.0	40.6	39.9	51.0	52.9
R.m.s. deviations						
Bond lengths (Å)	0.006	0.006	0.006	0.006	0.007	0.004
Bond angles (°)	0.801	0.776	0.812	0.842	1.224	0.616

*Values in parentheses are for the highest-resolution shell.

Supplementary Table 8. Primers used for molecular cloning and site-directed mutagenesis

Primers used for cloning the genes into the pCya vector

Ldum_LeSH1a	Forward (BamHI)	GAAGAAGGATCCGGATCAAAAGCAGAGTCTAAGGTT
	Reverse (PstI)	GGTGGTCTGCAGCTAGAGATTATTCACACTGCTTAG
Lani_LeSH1b	Forward (BamHI)	GAAGAAGGATCCGACCCTAAGCAGAACAACGAGCCA
	Reverse (SalI)	GGTGGTGTCGACTCACCGGGCTTTGTGAGTATTCAC
Ldum_LeSH2	Forward (BamHI)	GAAGAAGGATCCGAAGAGCGTTTAGAAAGATC
	Reverse (PstI)	GGTGGTCTGCAGTTATAACTCACTATTGTAGC
Lp_LeSH2	Forward (BamHI)	GAAGAAGGATCCGAGTCAAAATCAGAAGTACA
	Reverse (PstI)	GGTGGTCTGCAGTTAAAAATTAGTATATCTGTTC
Lani_LeSH2a	Forward (BamHI)	GAAGAAGGATCCCAATCCAAGAACGAGTCCTCCTCC
	Reverse (PstI)	GGTGGTCTGCAGTCACAGTTTGCTGTAGCGGTTGTT
Lani_LeSH2b	Forward (BamHI)	GAAGAAGGATCCGAGTCCAAGAAGGAGAACTC
	Reverse (PstI)	GGTGGTCTGCAGTCAAAAGCTTGTGTAGCGGTTCTT
Ldum_LUSH	Forward (BamHI)	GAAGAAGGATCCCGATTTAAGATAGAAGAAAC
	Reverse (PstI)	GGTGGTCTGCAGTTACATTAGGGGTGTTTTTGAAGA
Lp_DoSH	Forward (BamHI)	GAAGAAGGATCCACGATAGAATGTTATTCTATTG
	Reverse (PstI)	GGTGGTCTGCAGTTAAATATTTTTGCAAAGAGTCGGT
Lp_RavO	Forward (BamHI)	GTTGTTGGATCCCCTACTGGAATCGTATTAAG
	Reverse (SphI)	GGTGGTGCATGCTTAGCCTCCTTTGGTTGGATTTCG
Llo_LeSH	Forward (BamHI)	GTTGTTGGATCCGAAGCCATGCAGAAGAACGAGCTG
	Reverse (XbaI)	GGTGGTTCTAGATTAAACGTAATCTGTAAAGTGTT
Llo_LUSH	Forward (BamHI)	GTTGTTGGATCCCAGAGCAAGCTGGAGGAAGCCGACA
	Reverse (SphI)	GGTGGTGCATGCTTAAAAGTTTTTGCAACTCTGCTGG

Primers used for cloning the genes into the pETM11/pETM30 vector

Ldum_LUSH	Forward (NcoI)	GGTGGTCCATGGGATTTAAGATAGAAGAAACCCGC
	Reverse (BamHI)	GTTGTTGGATCCTTACATTAGGGGTGTTTTTGAAGAAGAATT
Lp_DoSH C-SH2	Forward (NcoI)	GGTGGTCCATGGAGAAGGAAGCCGAAACCAAC
	Reverse (BamHI)	GTTGTTGGATCCTCAGATGTTTTTACACAATGT

Site-directed mutagenesis primers

Llo_LeSH R46K	Forward	CAAGGCAGTGTTCCTCATCAAAGATAGCTCTACATATCCAG
	Reverse	CTGGATATGTAGAGCTATCTTTGATGAGGAACACTGCCTTG
Llo_LeSH R71L	Forward	GATATAGTCAAGCACATACTGTTTGGCTTGACCGACAAAG
	Reverse	CTTTGTCGGTCAAGCCAAACAGTATGTGCTTGACTATATC
Llo_LeSH P85A	Forward	GAAAACGGCACCCAAAGCCCCTCATGAGCCATTG
	Reverse	CAATGGCTCATGAGGGGCTTTGGGTGCCGTTTTC
Ldum_LeSH1 D44S	Forward	GTTTCTGATTCGCGAAAGCGAGAAGTTGCAGGG
	Reverse	CCCTGCAACTTCTCGCTTTCGCGAATCAGAAAC
Lp_RavO R266K	Forward	CAAAGCCGAGTTTATCCTCAAATGGTCCTCAGCCAACGAG
	Reverse	CTCGTTGGCTGAGGACCATTTGAGGATAAACTCGGCTTTG
Lp_RavO T310A	Forward	GAACTGTTTAGCATCAACGCCGCTTCTAACAAGGTGAC
	Reverse	GTCACCTTGTTAGAAGCGGCGTTGATGCTAAACAGTTC
