# Supplementary material for: Identification and characterization of a large family of superbinding bacterial SH2 domains
Source: Nat Commun. 2018 Oct 31;9:4549. doi: 10.1038/s41467-018-06943-2 (PMC6208348; doi:10.1038/s41467-018-06943-2)
Supplement: Supplementary file 3 — Reporting Summary [file 41467_2018_6943_MOESM3_ESM.pdf]

nature research

Corresponding author(s): Shawn Li, NCOMMS-18-05607A

Reporting Summary

Nature Research wishes to improve the reproducibility of the work that we publish. This form provides structure for consistency and transparency in reporting. For further information on Nature Research policies, see Authors & Referees and the Editorial Policy Checklist.

Statistical parameters

When statistical analyses are reported, confirm that the following items are present in the relevant location (e.g. figure legend, table legend, main text, or Methods section).

n/a	Confirmed	
☐	☒	The exact sample size (n) for each experimental group/condition, given as a discrete number and unit of measurement
☐	☒	An indication of whether measurements were taken from distinct samples or whether the same sample was measured repeatedly
☐	☒	The statistical test(s) used AND whether they are one- or two-sided<br>*Only common tests should be described solely by name; describe more complex techniques in the Methods section.*
☒	☐	A description of all covariates tested
☒	☐	A description of any assumptions or corrections, such as tests of normality and adjustment for multiple comparisons
☐	☒	A full description of the statistics including central tendency (e.g. means) or other basic estimates (e.g. regression coefficient) AND variation (e.g. standard deviation) or associated estimates of uncertainty (e.g. confidence intervals)
☐	☒	For null hypothesis testing, the test statistic (e.g. F, t, r) with confidence intervals, effect sizes, degrees of freedom and P value noted<br>*Give P values as exact values whenever suitable.*
☒	☐	For Bayesian analysis, information on the choice of priors and Markov chain Monte Carlo settings
☒	☐	For hierarchical and complex designs, identification of the appropriate level for tests and full reporting of outcomes
☒	☐	Estimates of effect sizes (e.g. Cohen's d, Pearson's r), indicating how they were calculated
☐	☒	Clearly defined error bars<br>*State explicitly what error bars represent (e.g. SD, SE, CI)*

Our web collection on statistics for biologists may be useful.

Software and code

Policy information about availability of computer code

Data collection	No software was used
Data analysis	all software names are included in the manuscript with references: crystallographic software (iMOSFLM, SCALA, AutoRickhshaw server, Phenix, Coot, CCP4 suite, HKL3000), structure analysis software/server (DALI, pymol, PISA server, ElNemo), sequence analysis software/ server (STRAP, MultiProt, MAFFT, SMART, Pfam, HMM suite, Jalview, TwoSampleLogo, SMALI, NCBI BLAST, PsiPred)

For manuscripts utilizing custom algorithms or software that are central to the research but not yet described in published literature, software must be made available to editors/reviewers upon request. We strongly encourage code deposition in a community repository (e.g. GitHub). See the Nature Research guidelines for submitting code & software for further information.

Data

Policy information about availability of data

All manuscripts must include a data availability statement. This statement should provide the following information, where applicable:

- Accession codes, unique identifiers, or web links for publicly available datasets
- A list of figures that have associated raw data
- A description of any restrictions on data availability

Atomic coordinates and structure factors have been deposited in the Protein Data Bank (PDB) with accession numbers 6E8H (LeSH), 6E8I (LeSH-phosphotyrosine

complex), 6E8M (LeSH-DnaJ-A1 pTyr381 peptide complex), 6E8K (LeSH-IL2Rβ pTyr387 peptide complex), 6DM3 [http://dx.doi.org/10.2210/pdb6DM3/pdb] (RavO SH2 domain), and 6DM4 [http://dx.doi.org/10.2210/pdb6DM4/pdb] (RavO SH2 domain-Shc1 pTyr317 peptide complex). All other data are available upon request.

Field-specific reporting

Please select the best fit for your research. If you are not sure, read the appropriate sections before making your selection.

☒ Life sciences ☐ Behavioural & social sciences ☐ Ecological, evolutionary & environmental sciences

For a reference copy of the document with all sections, see nature.com/authors/policies/ReportingSummary-flat.pdf

Life sciences study design

All studies must disclose on these points even when the disclosure is negative.

Sample size	Three replicate experiments were performed for the infection studies, that produced statistically significant differences evaluated by two-tailed Student's paired t-test, as described in Supplementary Fig. 5, and therefore we concluded that the sample size was sufficient.
Data exclusions	No data were excluded
Replication	Two biological replicate experiments were conducted for a part of infection studies, as shown in Supplementary Fig. 5a and 5b, and the trends were reproducible.
Randomization	N/A
Blinding	N/A

Reporting for specific materials, systems and methods

Materials & experimental systems

n/a	Involved in the study
☒	☐ Unique biological materials
☐	☒ Antibodies
☐	☒ Eukaryotic cell lines
☒	☐ Palaeontology
☒	☐ Animals and other organisms
☒	☐ Human research participants

Methods

n/a	Involved in the study
☒	☐ ChIP-seq
☒	☐ Flow cytometry
☒	☐ MRI-based neuroimaging

Antibodies

Antibodies used	4G10 Platinum mouse anti-pTyr antibody (1:3000 dilution, Millipore, catalog # 05-1050), mouse anti-SHC1 antibody (1:4000 dilution, BD Biosciences, catalog # 610878), rabbit anti-VCP antibody (1:10000 dilution, Abcam, catalog # ab109240), goat anti-mouse IgG-HRP conjugate (1:3000 dilution, Bio-Rad, catalog # 170-6516), goat anti-rabbit IgG-HRP conjugate (1:3000 dilution, Bio-Rad, catalog # 170-6515), mouse anti-CyaA antibody (Santa Cruz Biotechnology, catalog # sc-13582) diluted 1:5000.
Validation	All antibodies are commercially available and validation images are provided in the company websites.

Eukaryotic cell lines

Policy information about cell lines

Cell line source(s)	From ATCC (U937 cell line, Catalog # ATCC CRL-1593.2)
Authentication	From ATCC
Mycoplasma contamination	The cell line was tested negative with mycoplasma contamination
Commonly misidentified lines (See ICLAC register)	N/A
